# Dengue virus nonstructural protein 1 activates platelets via Toll-like receptor 4, leading to thrombocytopenia and hemorrhage

**DOI:** 10.1371/journal.ppat.1007625

**Published:** 2019-04-22

**Authors:** Chiao-Hsuan Chao, Wei-Chueh Wu, Yen-Chung Lai, Pei-Jane Tsai, Guey-Chuen Perng, Yee-Shin Lin, Trai-Ming Yeh

**Affiliations:** 1 Institute of Basic Medical Sciences, College of Medicine, National Cheng Kung University, Tainan, Taiwan; 2 Department of Medical Laboratory Science and Biotechnology, College of Medicine, National Cheng Kung University, Tainan, Taiwan; 3 Department of Microbiology and Immunology, College of Medicine, National Cheng Kung University, Tainan, Taiwan; Purdue University, UNITED STATES

## Abstract

Dengue virus (DENV) infection, the most common mosquito-transmitted viral infection, can cause a range of diseases from self-limiting dengue fever to life-threatening dengue hemorrhagic fever and shock syndrome. Thrombocytopenia is a major characteristic observed in both mild and severe dengue disease and is significantly correlated with the progression of dengue severity. Previous studies have shown that DENV nonstructural protein 1 (NS1), which can be secreted into patients’ blood, can stimulate immune cells via Toll-like receptor 4 (TLR4) and can cause endothelial leakage. However, it is unclear whether DENV NS1 can directly induce platelet activation or cause thrombocytopenia during DENV infection. In this study, we first demonstrated that DENV but not Zika virus cell culture supernatant could induce P-selectin expression and phosphatidylserine (PS) exposure in human platelets, both of which were abolished when NS1 was depleted from the DENV supernatant. Similar results were found using recombinant NS1 from all four serotypes of DENV, and those effects were blocked in the presence of anti-NS1 F(ab’)_2_, anti-TLR4 antibody, a TLR4 antagonist (*Rhodobacter sphaeroides* lipopolysaccharide, LPS-Rs) and a TLR4 signaling inhibitor (TAK242), but not polymyxin B (an LPS inhibitor). Moreover, the activation of platelets by DENV NS1 promoted subthreshold concentrations of adenosine diphosphate (ADP)-induced platelet aggregation and enhanced platelet adhesion to endothelial cells and phagocytosis by macrophages. Finally, we demonstrated that DENV-induced thrombocytopenia and hemorrhage were attenuated in TLR4 knockout and wild-type mice when NS1 was depleted from DENV supernatant. Taken together, these results suggest that the binding of DENV NS1 to TLR4 on platelets can trigger its activation, which may contribute to thrombocytopenia and hemorrhage during dengue infection.

## Author summary

Over the past 50 years, dengue has been a continuing global threat, with no effective vaccine or specific antiviral drug. Dengue infection causes a wide range of outcomes, from fever-like symptoms to severe dengue hemorrhagic fever. Thrombocytopenia, a reduction in platelet count, is a common feature observed in both mild and severe dengue and is correlated with disease severity. In this study, we used dengue viral supernatant or DENV recombinant NS1 protein to stimulate human-isolated platelets. We found that DENV NS1 could directly activate platelets through TLR4 and could further enhance platelet aggregation, adhesion to endothelial cells and phagocytosis by macrophages, which could lead to thrombocytopenia. We also proved that both NS1 and TLR4 are critical for DENV-induced thrombocytopenia and hemorrhage using a DENV-induced hemorrhagic mouse model. Our study reveals a new pathogenic role of NS1 during dengue infection and highlights that NS1 should be a topic of attention in the development of therapeutic drugs and vaccines against dengue infection.

## Introduction

Dengue is the most widespread mosquito-borne viral infection, infecting approximately 390 million people and causing 500,000 hospitalizations every year [1, 2]. Most infected patients have a mild, self-limited disease known as dengue without warning signs, but some can develop severe dengue, which is characterized by plasma leakage, fluid accumulation, severe bleeding, and organ impairment [3]. The underlying mechanisms by which mild dengue progresses to severe dengue are still unclear [4–6]. Thrombocytopenia, a common feature observed in both mild and severe dengue disease, is correlated with disease severity and is considered a predictive biomarker of severe dengue [7, 8]. However, the mechanisms that cause thrombocytopenia during dengue infection are not fully understood.

Previous studies have demonstrated that elevated surface P-selectin and increased phosphatidylserine (PS) exposure among platelets are correlated with thrombocytopenia in dengue patients [9, 10]. The underlying mechanism of platelet activation has been analyzed by proteomic analysis of dengue patients’ platelets, and circulating histones in dengue patients’ plasma have been suggested to activate platelets [9]. In addition, dengue virus (DENV) infection can induce platelet activation and apoptosis [10]. However, it remains unclear which viral component, if any, participates in platelet activation and apoptosis.

Dengue nonstructural protein 1 (NS1) is a 48 kDa glycoprotein that can be expressed on the DENV-infected cell surface as a dimer and is the only viral protein secreted into the blood circulation, as a hexamer, in dengue patients [11]. The concentration of NS1 in the sera of DHF/DSS patients ranges from 0.01–50 μg/ml, which correlates with disease severity [5, 12]. Recently, an increasing number of studies have shown that NS1 plays a critical role in dengue pathogenesis both *in vitro* and *in vivo*, which includes enhancing DENV replication/infection, directly inducing vascular leakage, and causing cytokine release from immune cells [13–18]. Furthermore, a previous study indicated that relative levels of NS1 antigen in dengue patients’ sera were negatively correlated with platelet count [19]. Recently, Modhiran et al. demonstrated that Toll-like receptor 4 (TLR4), a well-known receptor of lipopolysaccharide (LPS), acts as the receptor of NS1 in immune cells [15, 20]. It is known that LPS can induce platelet activation and potentiate platelet aggregation via TLR4/MyD88 signal transduction [21]. Since both NS1 and LPS can activate cells through TLR4, in this study, we propose and test the hypothesis that NS1 can induce platelet activation and enhance aggregation through TLR4, leading to thrombocytopenia and hemorrhage during dengue infection.

## Results

### DENV NS1 contributes to DENV supernatant-induced platelet activation and apoptosis

**Fig 1:**
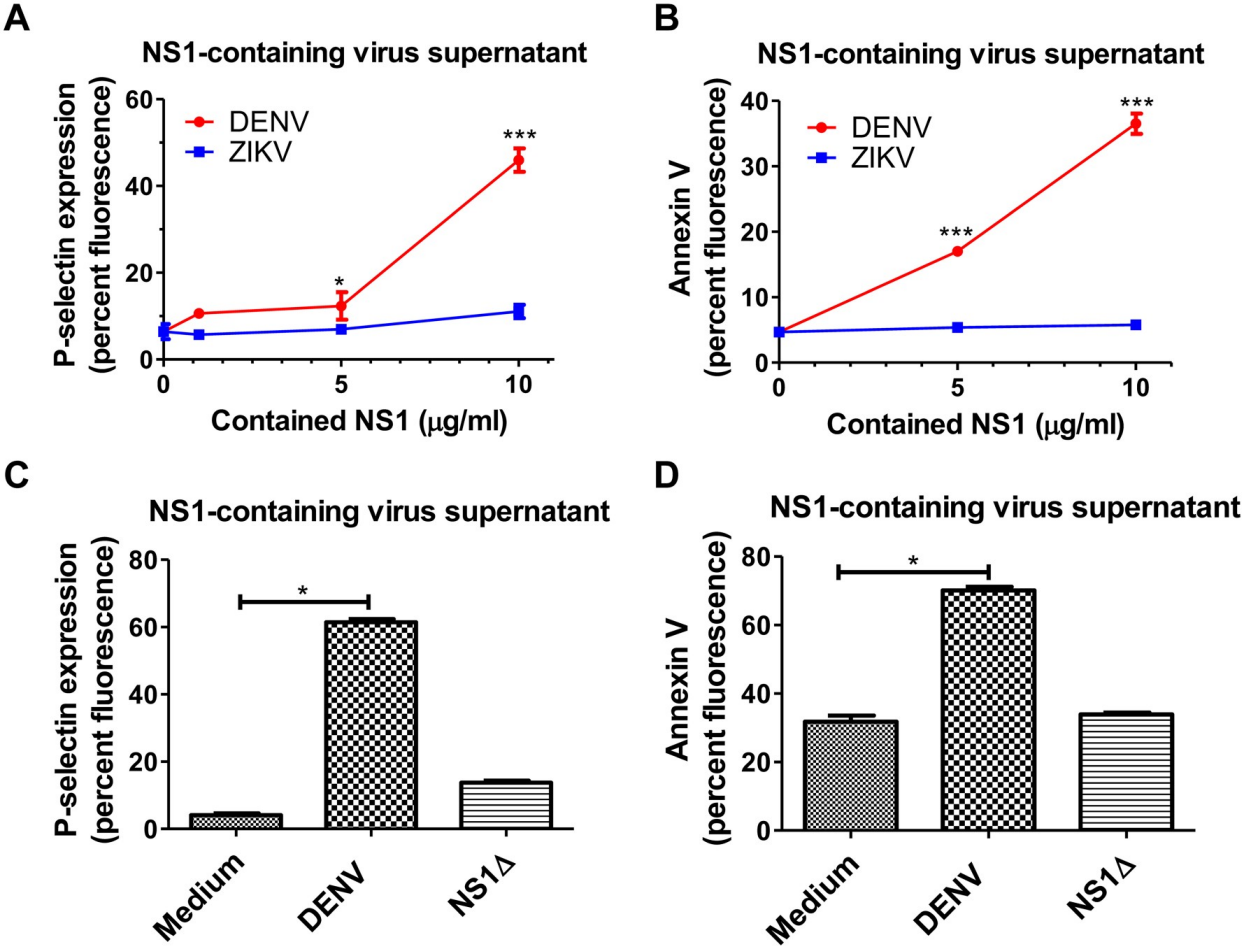
DENV NS1 is critical for DENV-induced P-selectin expression and PS exposure in platelets. **(A) (B)** Platelets were incubated for 1 h with DENV or ZIKV supernatant that contained different concentrations of NS1 as indicated (n = 4). **(C) (D)** Platelets were incubated with medium, 10 μg/ml NS1-containing DENV supernatant or NS1-depleted DENV supernatant (NS1Δ) for 1 h (n = 3). The percent fluorescence of P-selectin surface expression on platelets and annexin V binding to platelets were analyzed by FACSCalibur flow cytometry. *P<0.05, ***P<0.001; unpaired t-test (panels A and B), Kruskal-Wallis ANOVA (panels C and D).

To verify whether NS1 is involved in DENV-induced platelet activation, we incubated isolated human platelets with DENV supernatant containing different concentration of NS1 as indicated (S1 Fig). We found that DENV supernatant containing 5 μg/ml NS1 significantly induced the expression of a platelet activation marker (P-selectin, CD62P) and an apoptosis marker (PS) on the surfaces of platelets. However, Zika virus (ZIKV) supernatant containing a similar amount of NS1 did not show a significant effect on platelet activation or apoptosis (Fig 1A and 1B, S2A and S2B Fig). We also incubated platelets with DENV or ZIKV recombinant proteins; consistently, only DENV NS1, but not ZIKV NS1, could trigger platelet activation and apoptosis (S3 Fig). To further verify that NS1 is involved in DENV supernatant-induced platelet activation/apoptosis, we used agarose-conjugated anti-dengue NS1 antibody to remove NS1 from DENV supernatant. As anticipated, both surface P-selectin expression and PS exposure of platelets in response to DENV supernatant were attenuated when NS1 was depleted from the supernatant (Fig 1C and 1D, S2C and S2D Fig). These results indicate that DENV NS1 contributes to DENV-supernatant-induced platelet activation and apoptosis.

### DENV NS1 directly induces platelet activation and apoptosis

**Fig 2:**
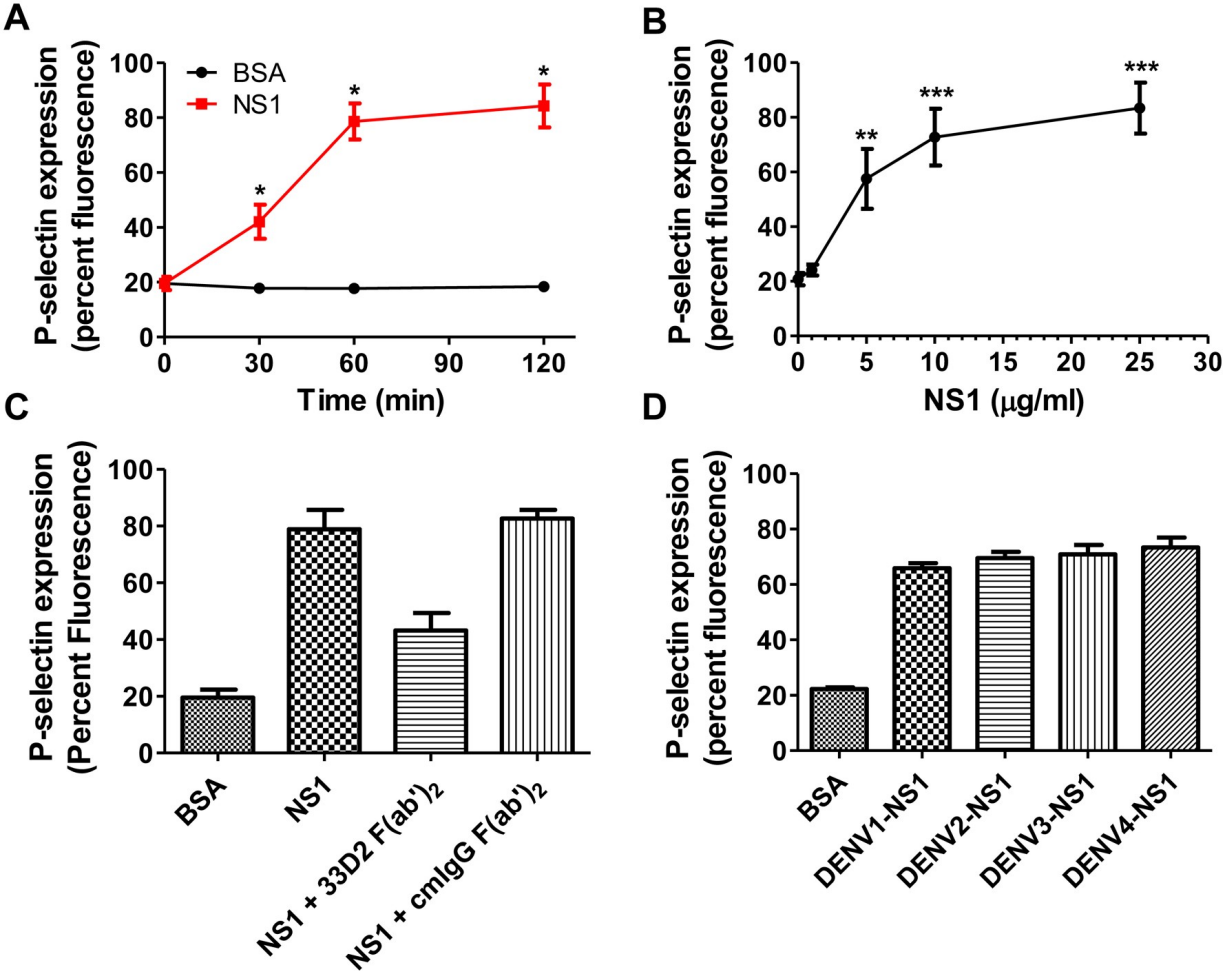
DENV NS1 directly induces platelet activation in a dose- and time-dependent manner. **(A)** Platelets were treated with BSA or DENV NS1 recombinant proteins (10 μg/ml) for the indicated time (n = 3). **(B)** Platelets were treated with different concentrations of DENV NS1 recombinant proteins for 1 h (n = 3). **(C)** Platelets were treated with BSA or DENV NS1 (10 μg/ml) (cotreated with or without 25 μg/ml anti-NS1 specific antibodies F(ab’)_2_ fragment, 33D2 F(ab’)_2_, or the isotype-matched mouse antibodies F(ab’)_2_ and cmIgG F(ab’)_2_) for 1 h (n = 3). **(D)** Platelets were treated with BSA or DENV1-4 NS1 (10 μg/ml) for 1 h (n = 3). The percent fluorescence of P-selectin surface expression on platelets was analyzed by FACSCalibur flow cytometry. ***P<0.001; unpaired t-test (panels A and B), Kruskal-Wallis ANOVA (panels C and D).

**Fig 3:**
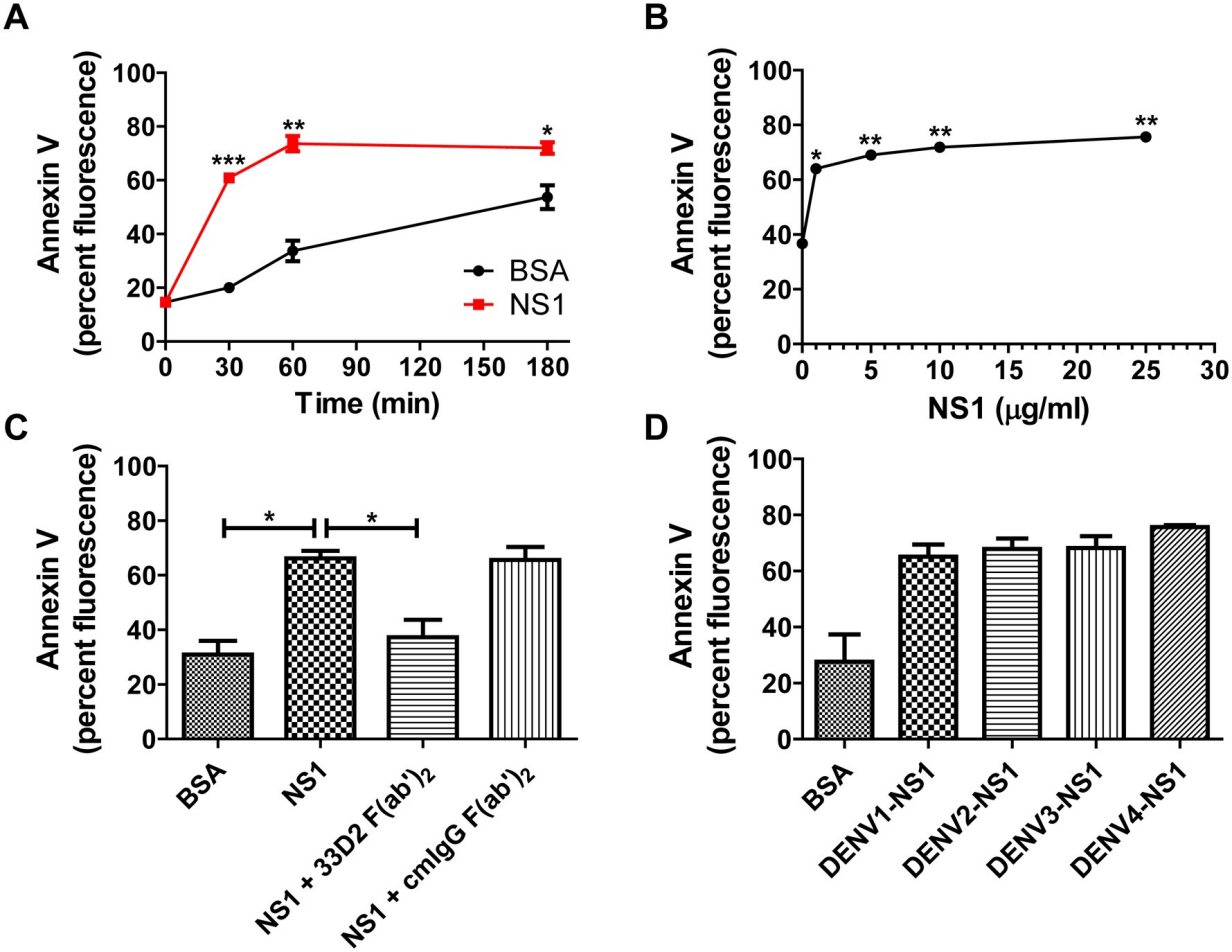
DENV NS1 directly induces platelet apoptosis in a dose- and time-dependent manner. **(A)** Platelets were treated with BSA or DENV NS1 recombinant protein (10 μg/ml) for the indicated time (n = 3). **(B)** Platelets were treated with different concentrations of DENV NS1 recombinant protein for 1 h (n = 3). **(C)** Platelets were treated with BSA or DENV NS1 (10 μg/ml) (cotreated with or without 25 μg/ml 33D2 F(ab’)_2_, or cmIgG F(ab’)_2_) for 1 h (n = 3). **(D)** Platelets were treated with BSA or DENV1-4 NS1 (10 μg/ml) for 1 h (n = 3). The percent fluorescence of annexin V binding to platelets was analyzed by FACSCalibur flow cytometry. *P<0.05, **P<0.01, ***P<0.001; unpaired t-test (panels A and B), Kruskal-Wallis ANOVA (panels C and D).

To further confirm that NS1 could directly induce platelet activation, we incubated isolated platelets with different concentrations of DENV NS1 recombinant proteins or bovine serum albumin (BSA). We found that DENV NS1 induced a maximal increase in surface P-selectin expression at 1 h after stimulation (Fig 2A and S4A Fig) and that the magnitude of the increase depended on the dose of NS1 (1–25 μg/ml) (Fig 2B and S4B Fig). In addition, the anti-NS1 neutralizing monoclonal antibody (mAb) 33D2, which recognizes a conserved NS1 wing domain region and is able to block the effect of DENV NS1 both *in vitro* and *in vivo*, [22] was used to verify that the increase in P-selectin expression was indeed triggered by DENV NS1. To avoid Fc receptor-mediated platelet activation, we used the 33D2 F(ab’)_2_ fragment [23]. The results showed that cotreatment of NS1 with the 33D2 F(ab’)_2_ fragment attenuated the increase in platelet P-selectin expression induced by DENV NS1. However, the isotype-matched control mouse IgG F(ab’)_2_ fragment, used here as a negative control, did not block DENV NS1-induced platelet activation (Fig 2C and S4C Fig). In addition, all four serotypes of DENV NS1 were able to induce platelet activation (Fig 2D and S4D Fig). Furthermore, the apoptosis marker of interest (PS exposure on platelets) was also increased after 0.5 h of incubation with DENV NS1, reaching a maximum at 1 h after stimulation (Fig 3A and S5A Fig) in a dose-dependent manner (Fig 3B and S5B Fig). In addition, cleaved caspase 3, another apoptosis marker, was also increased after 0.5 h of DENV NS1 treatment (S6 Fig). The increased PS exposure induced by DENV NS1 was significantly inhibited by the 33D2 F(ab’)_2_ fragment but not by the isotype-matched control mouse IgG F(ab’)_2_ fragment (Fig 3C and S5C Fig). All four serotypes of DENV NS1 also increased PS exposure on platelets (Fig 3D and S5D Fig). These results suggest that DENV NS1 can directly trigger platelet activation and apoptosis.

### DENV NS1 binds to platelets and activates platelets through TLR4 signal transduction

**Fig 4:**
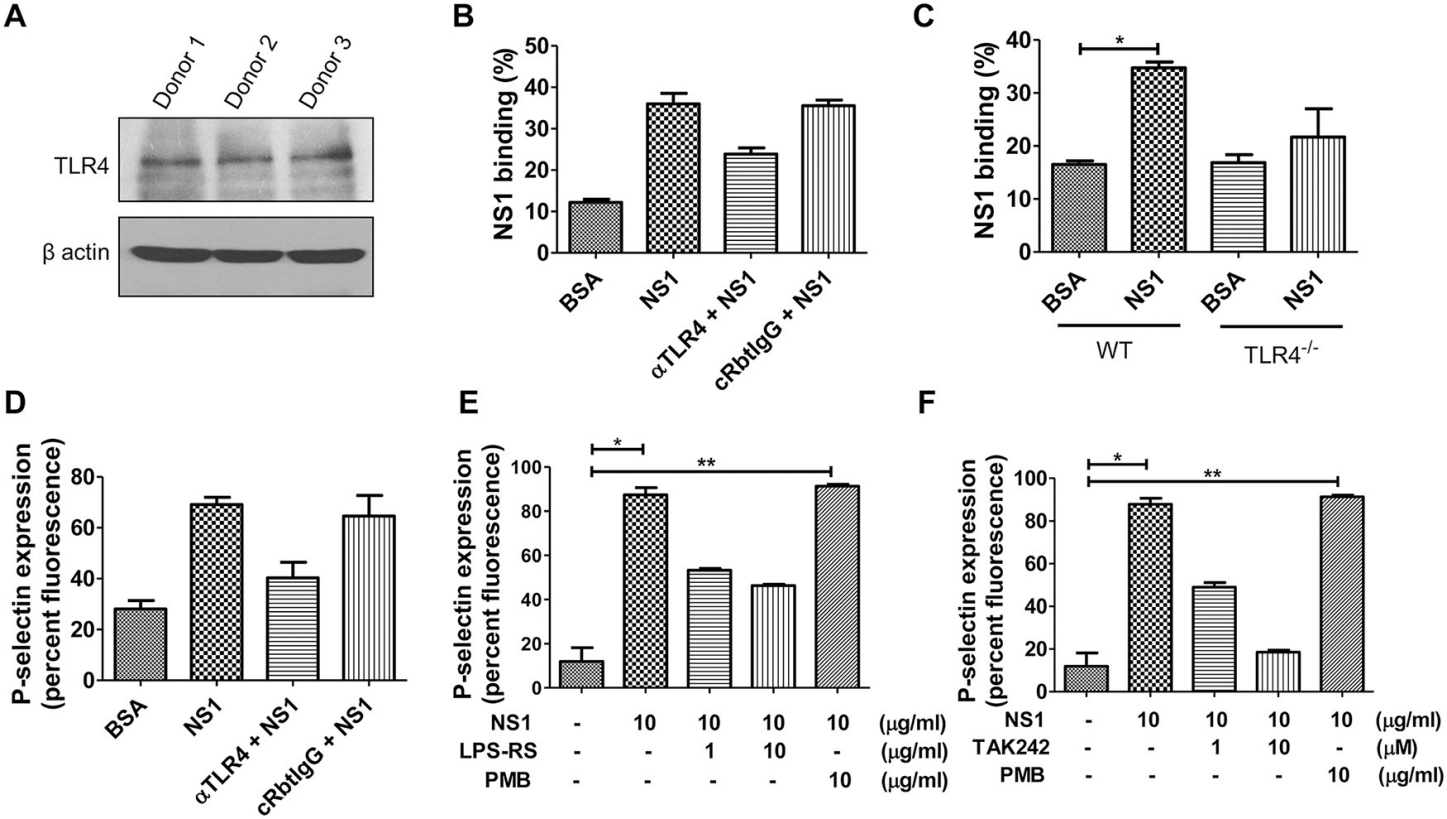
DENV NS1 binds to platelets and induces activation through TLR4 signal transduction. **(A)** The protein expression levels of TLR4 and β actin, an internal control, in human-isolated platelets from 3 different donors were detected using Western blotting (50 μg protein/lane). **(B)** Human-isolated platelets were preincubated with αTLR4 or a control Rabbit IgG for 1 h, and the binding of NS1 on platelet surfaces was determined by flow cytometry using FITC-conjugated anti-NS1 monoclonal antibodies (33D2-FITC) (n = 3). **(C)** The binding of NS1 on platelets isolated from wild-type or TLR4 knockout mice was determined by flow cytometry (n = 4). Platelets were preincubated with or without different concentrations of **(D)** αTLR4 **(E)** the TLR4 antagonist LPS-Rs, **(F)** the TLR4 signaling inhibitor TAK242, or the LPS inhibitor polymyxin B (10 μg/ml) for 30 min, followed by BSA or NS1 (10 μg/ml) stimulation for 1 h (n = 4). The percent fluorescence of NS1 binding and the P-selectin surface expression on platelets were analyzed by FACSCalibur flow cytometry. *P<0.05, **P<0.01; Kruskal-Wallis ANOVA (panels B to panel F).

Previous studies have shown that DENV NS1, acting through TLR4, induces the secretion of proinflammatory cytokines by immune cells [15, 20]. Accordingly, we investigated whether DENV NS1-triggered platelet activation is also mediated by TLR4. To assess the binding of NS1 on the platelet surface, we exposed platelets to DENV NS1 in buffer containing 0.01% NaN_3_ for 1 h at 4°C before fixation. The binding of NS1 was detected by anti-NS1 mAb 33D2 using both immunofluorescence assays and flow cytometry (S7 Fig). To study the role of TLR4 in NS1-induced platelet activation, we first confirmed the expression of TLR4 on human platelets (Fig 4A). Next, we used two approaches to verify the binding of NS1 to TLR4: using an anti-TLR4 antibody (αTLR4) to block the binding of NS1 to human-isolated platelets, and comparing the binding of NS1 between platelets from TLR4 knockout and wild-type mice. The results showed that the binding of NS1 to platelets was decreased in the presence of αTLR4 or in TLR4 knockout mice (Fig 4B and 4C, S8A and S8B Fig). Nevertheless, a previous study showed that DENV NS1 can also activate human immune cells via TLR2 and TLR6 [24]. Since TLR2 is also expressed on platelets, a direct enzyme-linked immunosorbent assay (ELISA) was performed to clarify whether NS1 can also bind to TLR2. Indeed, we found that NS1 could bind to both TLR4 and TLR2 but not BSA or an unrelated His-tag protein. However, the binding capacity of NS1 to TLR4 was stronger than that to TLR2 (S9 Fig). In addition, the involvement of TLR4 in NS1-induced platelet activation was confirmed by a TLR4 antagonist (*Rhodobacter sphaeroides* LPS, LPS-Rs) and a TLR4 signaling inhibitor (TAK242). Pretreatment of platelets with αTLR4, LPS-Rs or TAK242 attenuated NS1-induced surface P-selectin expression (Fig 4D, 4E and 4F, S8C, S8D and S8E Fig). Because all the NS1 recombinant proteins we used in this study contained very low LPS or endotoxin (0.036 EU/ml was found in 20 μg/ml NS1 recombinant protein), the result is unlikely to be due to bacterial LPS contamination in NS1 recombinant proteins [17]. In addition, to rule out the possibility of residual LPS in NS1 recombinant protein-induced activation of TLR4 signaling, we included an additional negative control group using DENV NS1 cotreated with the LPS-binding antibiotic polymyxin B (PMB). The results showed that PMB had no inhibitory effect on NS1-induced P-selection expression (Fig 4E and 4F, S8F Fig), even though all these inhibitors and αTLR4 could inhibit LPS-induced macrophage migration inhibitory factor (MIF) secretion in phorbol 12-myristate 13-acetate (PMA)-activated THP-1 cells (S8G Fig). Finally, we also compared the capacity of NS1 and LPS to induce P-selectin expression of platelets. Consistent with a previous study, LPS could induce P-selectin expression of platelets from 20% to approximately 40% when the concentration of LPS reached 50 μg/ml (S10 Fig) [25]. However, the presence of 10 μg/ml of NS1 was sufficient to increase the expression of P-selectin to approximately 80%. Taken together, these results suggest that DENV NS1 is much more potent than LPS for inducing platelet activation through TLR4 signal transduction.

### DENV NS1 enhances platelet aggregation in the presence of a subthreshold dose of adenosine diphosphate (ADP)

**Fig 5:**
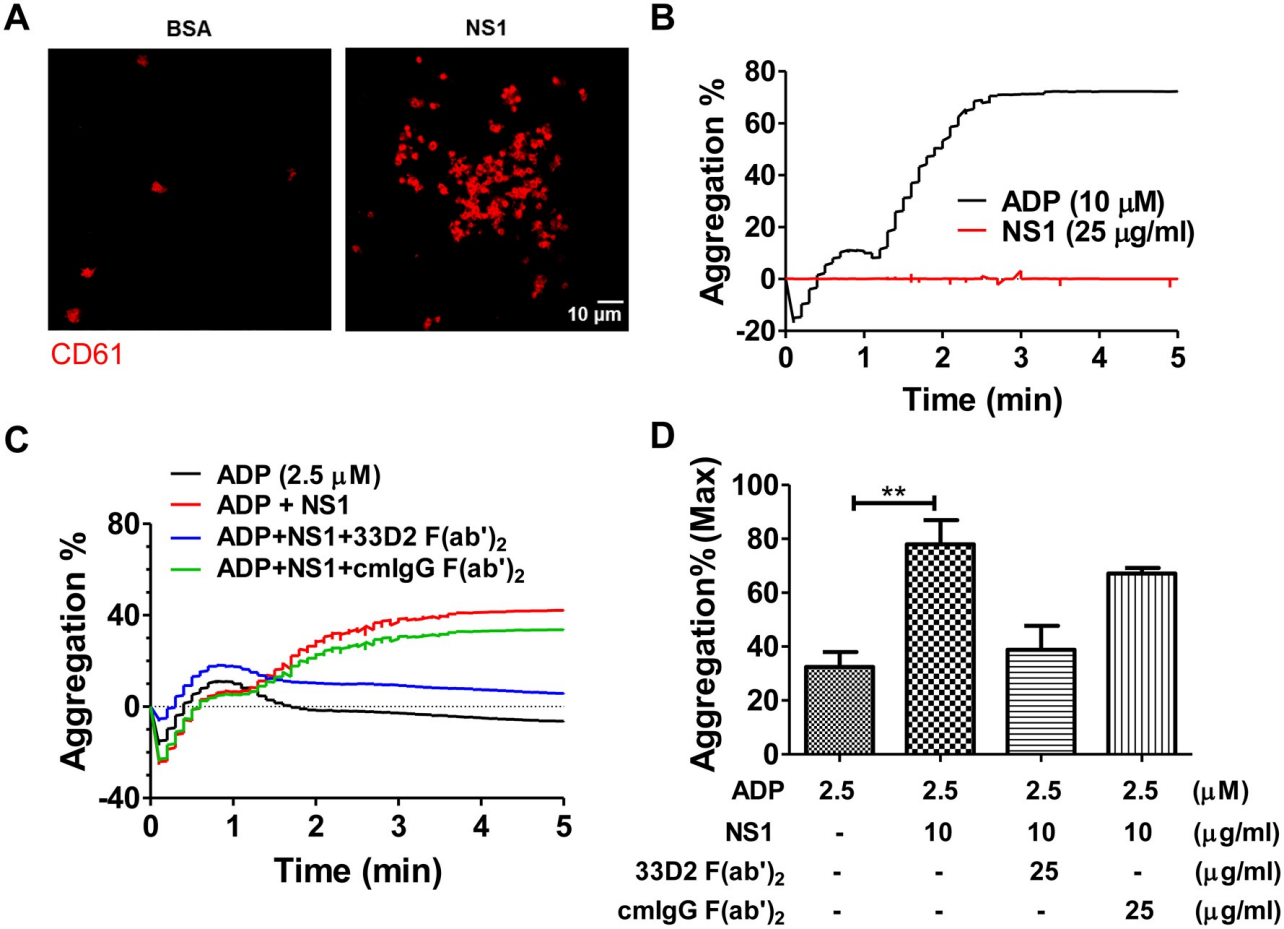
DENV NS1 enhances platelet aggregation ability after activation. **(A)** Platelets were plated on poly-L-lysine-coated coverslips and treated with BSA or DENV NS1 (10 μg/ml) for 1 h. After fixation and permeabilization, platelets were stained with anti-CD61 polyclonal antibody and an anti-rabbit Alexa 594-conjugated antibody. **(B)** The light transmission of platelet-rich plasma (PRP) was measured in a Chrono-log aggregometer for 5 min after ADP (10 μM) or DENV NS1 (10 μg/ml) was added. **(C)** PRP was treated with BSA or DENV NS1 (10 μg/ml) (cotreated with or without 25 μg/ml 33D2 F(ab’)2, or cmIgG F(ab’)2) for 1 h and stimulated with ADP (2.5 μM). The light transmission of PRP was measured in a Chrono-log aggregometer, and **(D)** the maximum percentage of platelet aggregation was calculated by an AggRAM^™^ platelet aggregation analyzer (n = 3). **P<0.01; Kruskal-Wallis ANOVA (panel D).

We found that NS1 could trigger platelet activation through TLR4, and it is known that LPS could induce platelet activation and potentiate platelet aggregation via TLR4/MyD88 signal transduction. Accordingly, we decided to investigate whether NS1 could enhance platelet aggregation as well. As shown in Fig 5A, platelets assumed a cluster-like morphology after NS1 stimulation. However, unlike the platelet agonist ADP, NS1 alone failed to induce platelet aggregation even at concentrations up to 25 μg/ml (Fig 5B). Nevertheless, when a subthreshold concentration of ADP (2.5 μM) was added to the NS1-incubated platelet-rich plasma (PRP), a significant enhancement of platelet aggregation was found; this increase could be rescued by the 33D2 F(ab’)_2_ fragment but not by the isotype-matched control mouse IgG F(ab’)_2_ fragment (Fig 5C and 5D). Since activated platelets can secrete ADP, we also measured the ADP secretion of platelets after NS1 stimulation. The results showed that NS1 could induce the ADP secretion of platelets in a time-dependent manner, and the enhancement of platelet aggregation induced by NS1 could be blocked by inhibitors of ADP receptors, such as BPTU (a P2Y_1_ inhibitor) and Clopidogrel (a P2Y_12_ inhibitor) (S11 Fig). These results may explain why NS1 activation can enhance platelet aggregation in the presence of a subthreshold dose of ADP.

### DENV NS1 increases platelet adherence to endothelial cells and phagocytosis by macrophages

**Fig 6:**
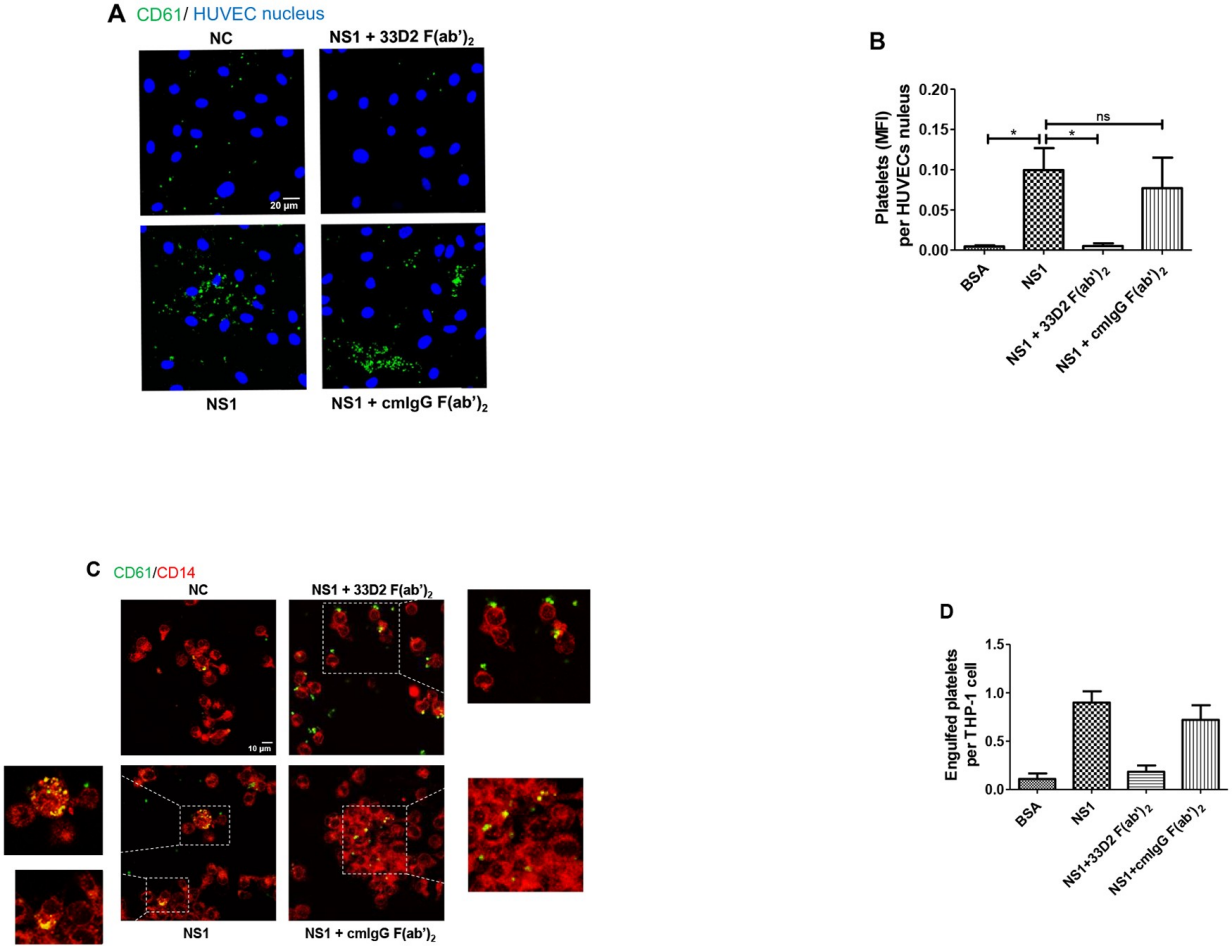
DENV NS1 increases platelet adherence to endothelial cells and phagocytosis by macrophages. **(A)** DENV-NS1-activated platelets were cultured with confluent HUVEC monolayers for 1 h, followed by washing and fixation. The adherent platelets were examined by confocal microscopy. CD61, a platelet marker, is stained in green, and HUVEC nuclei are stained with Hoechst (blue). **(B)** The adherent platelets on HUVEC monolayers in (A) were quantified in 100 HUVECs from three independent experiments and expressed as the mean fluorescence intensity (MFI) with ImageJ. **(C)** For the phagocytosis assay, DENV-NS1-activated platelets were cultured with PMA-activated THP-1 cells for 4 h. After fixation and permeabilization, the cells were stained with CD61 (green, a marker of platelets) and CD14 (red, a marker of monocytes/macrophages) and examined by confocal microscopy. The yellow dots represent the engulfed platelets, and the green dots represent the adherent platelets. **(D)** The average number of engulfed platelets per THP-1 cell was determined by counting 100 THP-1 cells per sample. The images were further acquired by confocal microscopy. *P<0.05; Kruskal-Wallis ANOVA (panels B and D).

Previous studies have indicated that the expression of P-selection on activated platelet surfaces increases the ability of platelets to bind to endothelial cells and leukocytes, and PS exposure on platelets designates them for phagocytosis by leukocytes [26–28]. To further investigate the fate of platelets after NS1 stimulation, we cocultured NS1-activated platelets with primary endothelial cells, human umbilical vein endothelial cells (HUVECs), and PMA-activated THP-1 cells. To exclude the effect of residual NS1 in platelet supernatant, we washed the platelets with Tyrode’s buffer before incubation with HUVECs. As shown in Fig 6A, we found an increase in the number of platelets adhering to HUVECs after NS1 stimulation compared with the BSA control group. The quantification of the mean fluorescence intensity (MFI) of CD61 expression of platelets per HUVECs was significantly increased, and the effect was significantly inhibited by the 33D2 F(ab’)_2_ fragment but not by the isotype-matched control mouse IgG F(ab’)_2_ fragment (Fig 6B). We also tested the endothelial permeability in the platelets-HUVECs coculture system. The endothelial permeability was increased 1 h after coincubation with washed NS1-treated platelets, but not BSA-treated platelets (S12 Fig). In the phagocytosis assay, the number of phagocytosed platelets (yellow dot) was also increased in the NS1-activated platelets compared with the BSA control group (Fig 6C) To quantify the results, we calculated the number of engulfed platelets per PMA-activated THP-1 cell. The number of engulfed platelets was significantly increased in the NS1-activation group, and cotreatment with the 33D2 F(ab’)_2_ fragment during NS1 stimulation prevented platelets from being phagocytosed by PMA-activated THP-1 (Fig 6D). In contrast, the isotype-matched control mouse IgG F(ab’)_2_ fragment did not significantly inhibit the phagocytosis of platelets by THP-1 cells (Fig 6D). In addition, NS1-activated platelets could also be engulfed by THP-1 cells without PMA activation (S13A Fig). This result suggests that NS1-activated platelets could trigger THP-1 activation. Therefore, we first tested the THP-1 differential profiles, and the results showed that coincubation of NS1-activated platelets could trigger the upregulation of monocyte chemoattractant protein 1 (MCP-1) mRNA expression in THP-1 cells but could not induce THP-1 cells to express the hallmarks of macrophages, such as cell adhesion and spread (S13B and S13C Fig) [29].

### DENV NS1 causes aggregated complex formation and cell death in the coculture system

**Fig 7:**
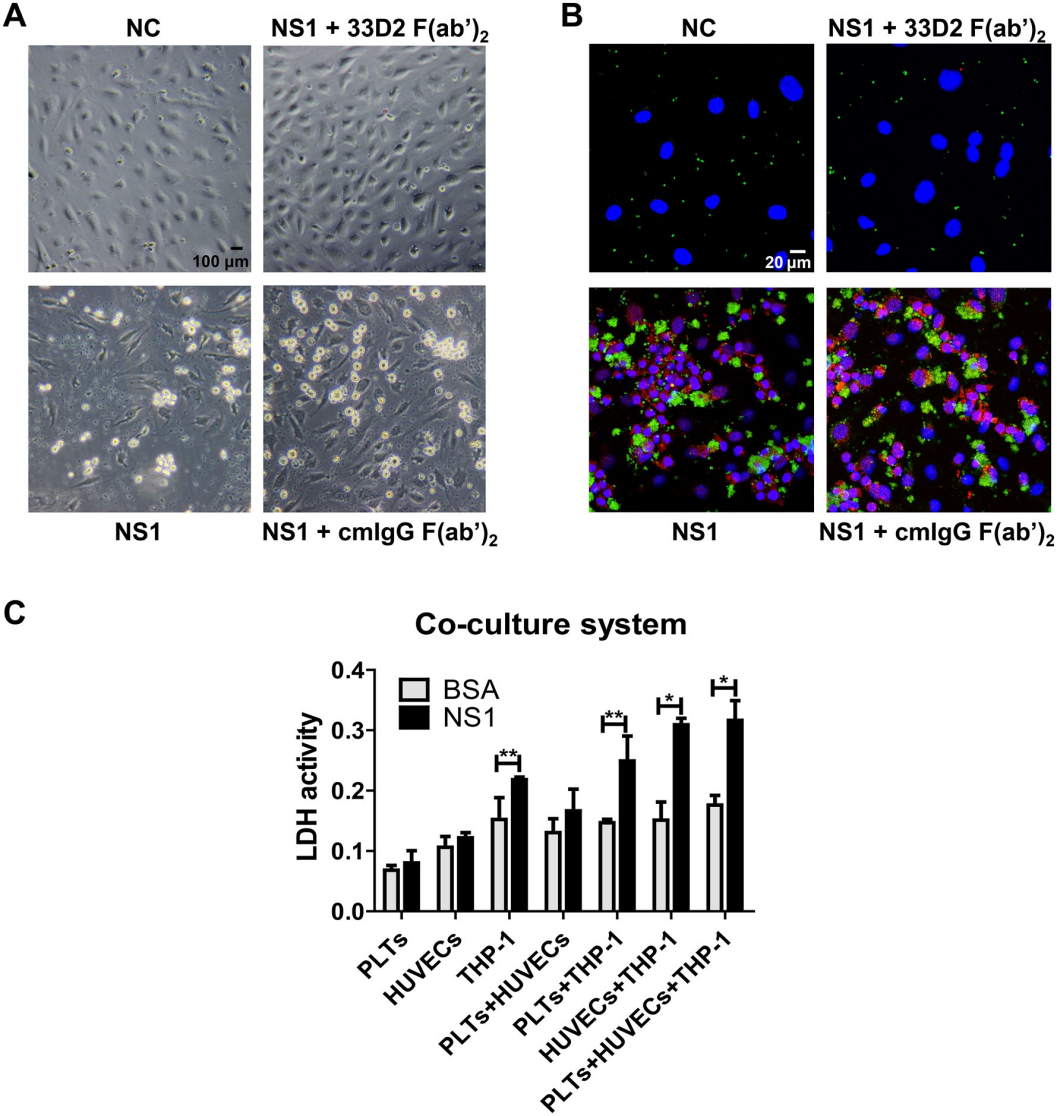
DENV NS1 causes the formation of aggregated complexes and cell death in a coculture system. **(A)** PMA-activated THP-1 cells and isolated platelets were added to confluent HUVECs on coverslips in 24-well plates with different treatments. After 1 h of incubation, unbound cells were washed out, and images were obtained by optical microscopy. **(B)** After fixation and permeabilization, monolayers were stained for CD61 (green), CD14 (red) and nuclei (blue). **(C)** Supernatants from different coculture conditions were collected, and cell death was determined using an LDH release assay. The images were further acquired by confocal microscopy. *P<0.05; **P<0.01; unpaired t-test (panels C).

To imitate the biological environment in a vessel, we established an *in vitro* coculture model containing isolated platelets from healthy donors, PMA-activated THP-1 cells, and a HUVEC monolayer. NS1 was added to the cocultured cells for 1 h, and the morphology and interaction of cells were observed. As expected, treatment with DENV NS1 induced both platelets and THP-1 cells to adhere to the HUVEC monolayer and triggered platelets to form clusters, which were inhibited in the presence of the 33D2 F(ab’)_2_ fragment but not the isotype-matched control mouse IgG F(ab’)_2_ fragment (Fig 7A and 7B). We also measured cell death with an LDH assay in the coculture system. Interestingly, NS1-induced LDH release could only occur when THP-1 cells were present (Fig 7C), indicating that NS1 stimulation can cause some THP-1 cells to die.

### NS1 and TLR4 play a crucial role in DENV-induced thrombocytopenia and hemorrhage in mice

**Fig 8:**
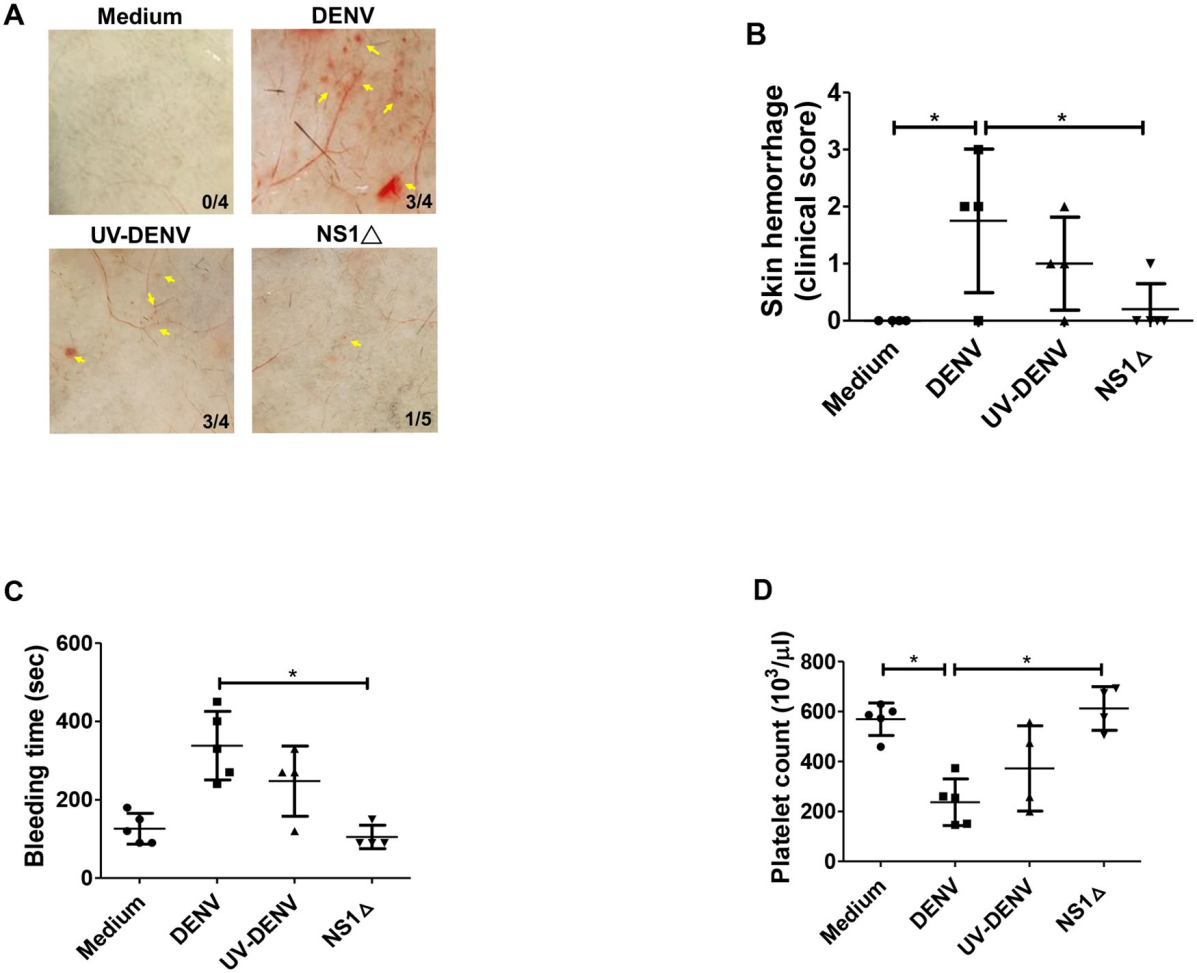
DENV NS1 is critical for DENV-induced thrombocytopenia, prolonged bleeding time, and hemorrhage in mice. A hemorrhagic C3H/HeN mouse model was created as described in the Methods. (A) Mouse skin samples were removed to observe local hemorrhage on day 3 after DENV injection. The number of mice with hemorrhage divided by the total number of mice inoculated in each group is indicated. Yellow arrows indicate local skin hemorrhage. (B) The clinical hemorrhage score was quantified and determined as digital hemorrhage severity. (C) The tail bleeding time and (D) platelet counts were also determined on day 3 before sacrifice. *P<0.05; Tukey’s multiple comparison test (panel B); Kruskal-Wallis ANOVA (panel C and D).

To test whether NS1 is involved in the pathogenesis of DENV-induced thrombocytopenia and hemorrhage *in vivo*, we used a previously established DENV-induced hemorrhagic C3H/HeN mouse model [30]. Inoculation of mice with high-titer DENV (2×10^8^ PFU/mouse) induced thrombocytopenia, prolonged bleeding time, and local hemorrhage in the skin of mice (Fig 8). To investigate the role of NS1 in the pathogenesis of thrombocytopenia and hemorrhage in mice, we compared the pathogenic outcomes after injecting mice with DENV, UV-inactivated DENV (UV-DENV), and NS1-depleted DENV (NS1Δ) supernatants. The viral titer and NS1 concentration in each DENV supernatant group are shown in S14 Fig. The clinical skin hemorrhage score was determined by the severity of hemorrhage, as shown in S15 Fig. Interestingly, we found that thrombocytopenia, prolonged bleeding time, and local skin hemorrhage induced by DENV were all significantly inhibited in mice inoculated with NS1-depleted DENV supernatant. Furthermore, these pathological changes were only partially prevented in mice inoculated with UV-DENV supernatant, which had no detectable viral titer but still contained 4.3 μg/ml NS1 (S14 Fig) (Fig 8). Since the secreted NS1 from infected mouse cells may also contribute to the thrombocytopenia-related pathogenesis, we collected mouse sera before sacrifice and measured the NS1 concentration using ELISA. The results showed that the circulating level of NS1 reached 200–300 ng/ml in mice inoculated with DENV. However, only 5–30 ng/ml of NS1 was detected in mice inoculated with UV-inactivated DENV, and 50–150 ng/ml of NS1 was detected in mice inoculated with NS1-depleted DENV (S16 Fig). These results indicated that the presence of NS1 in DENV supernatant may enhance DENV replication in mice, which, in turn, increases the secretion of NS1 to reach a concentration that can cause thrombocytopenia and hemorrhage in mice.

**Fig 9:**
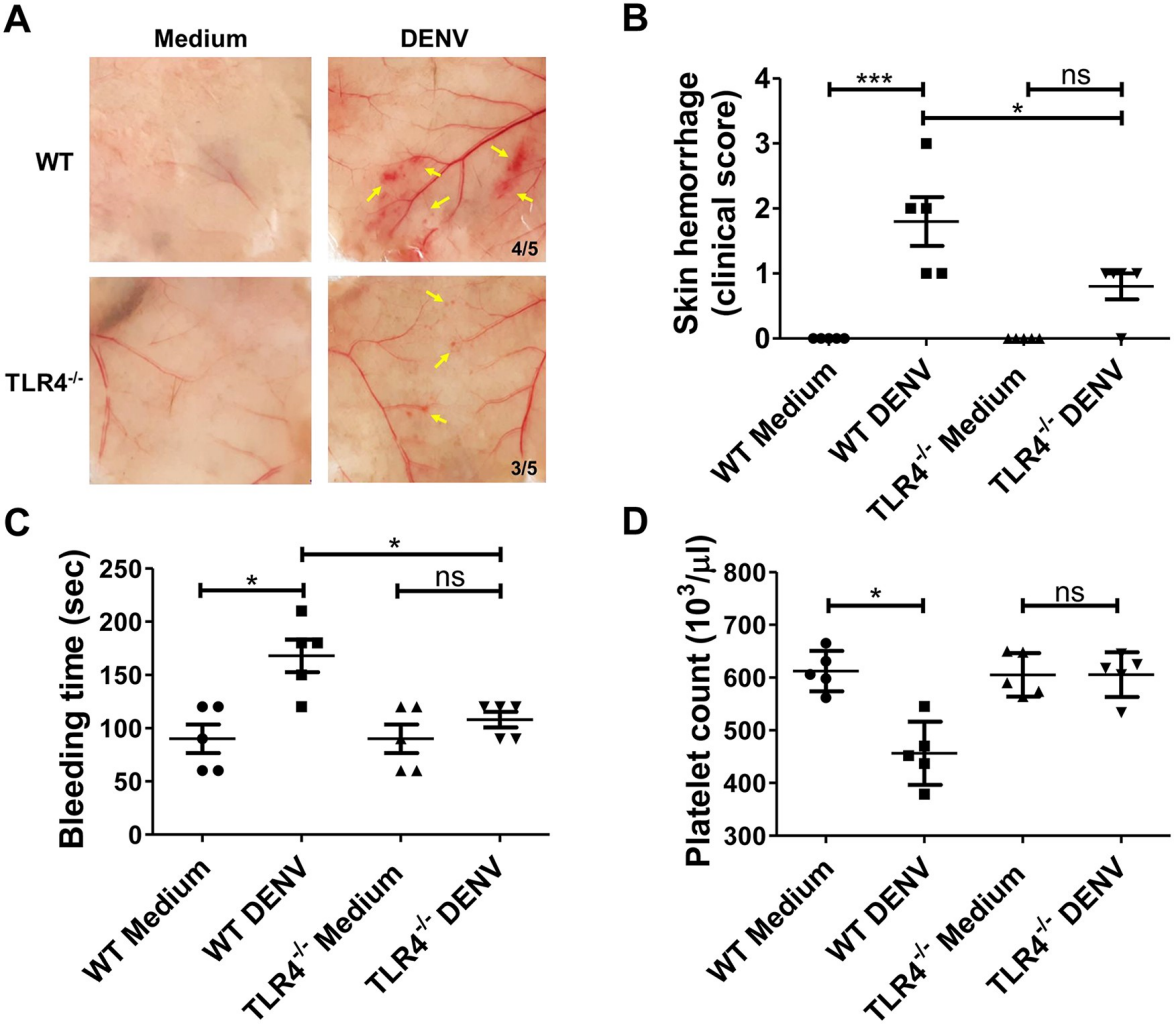
TLR4 is involved in DENV-induced prolongation of bleeding time and hemorrhage in mice. A hemorrhagic mouse model was performed with C57BL/6J mice (WT) and TLR4^-/-^ C57BL/6J background mice as described in the Methods. **(A)** Mouse skin samples were removed to observe local hemorrhage on day 3 after DENV injection. The number of mice with hemorrhage divided by the total number of mice inoculated in each group is indicated. Yellow arrows indicate local skin hemorrhage. **(B)** The clinical score of hemorrhage was quantified and determined as digital hemorrhage severity. **(C)** The tail bleeding time and **(D)** platelet counts were also determined on day 3 before sacrifice. *P<0.05, **P<0.01; Tukey’s multiple comparison test (panel B); Kruskal-Wallis ANOVA (panel C and D).

In addition, since we found that TLR4 might be the receptor through which NS1 activates platelets, we used TLR4 knockout mice (TLR4^-/-^) to investigate the role of TLR4 in DENV-induced hemorrhage in mice. As expected, we found that high-titer DENV could induce prolonged bleeding time, hemorrhage and thrombocytopenia in C57BL/6J wild-type mice but not in TLR4^-/-^ mice (Fig 9). Taken together, these results suggest that viral NS1 and host TLR4 proteins are critical for DENV-induced hemorrhage and thrombocytopenia during DENV infection.

## Discussion

**Fig 10:**
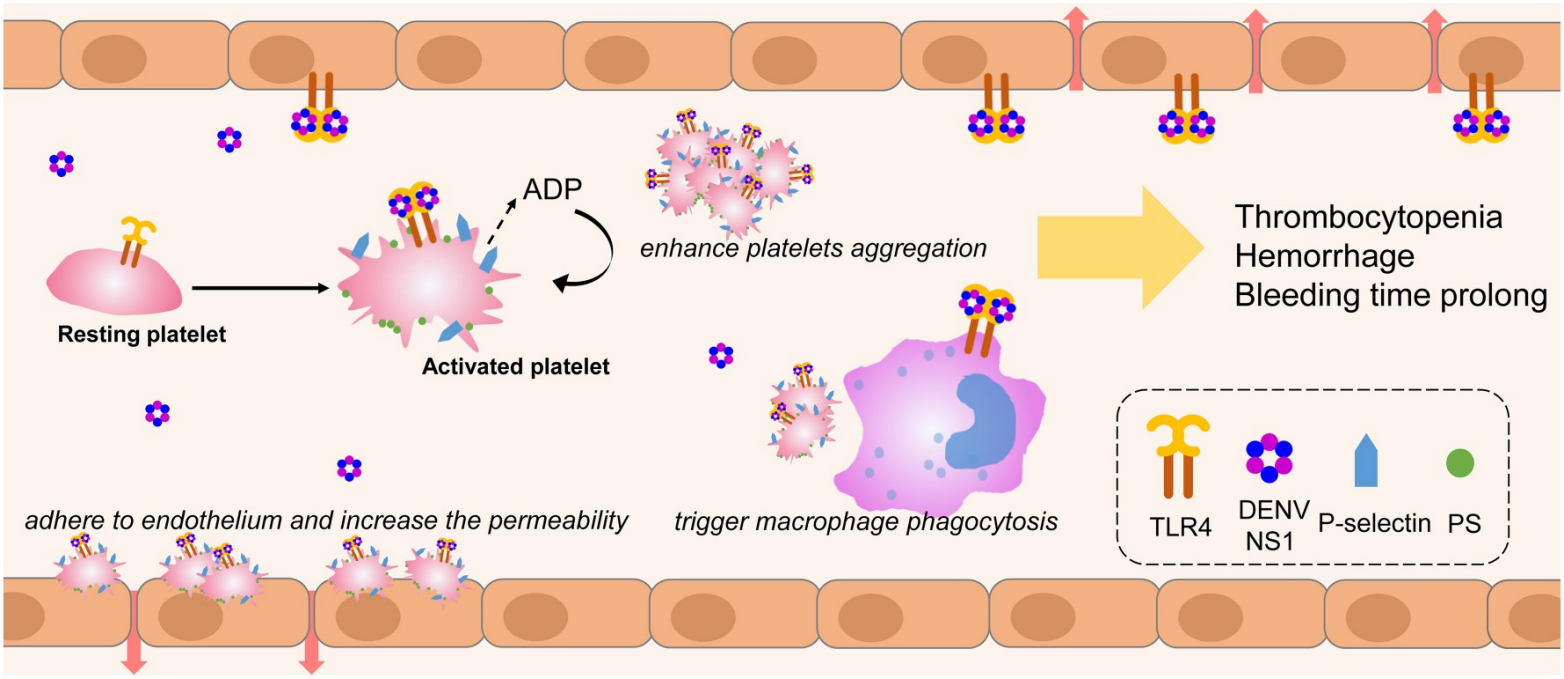
Proposed mechanisms of the contribution of DENV NS1 to cause thrombocytopenia and hemorrhage during DENV infection. Circulating DENV NS1 binds to platelets via TLR4 or other molecules to induce the release of ADP, which in turn elevates P-selectin expression and PS exposure on platelet surfaces leading to platelet activation and enhancement of the platelet aggregation. In addition, NS1-activated platelets are prone to adhere onto endothelium or phagocytosis by macrophages. On the other hand, NS1 can also bind to endothelial cells and macrophage to cause their activation and cytokine release. All these effects induced by NS1 can contribute to the thrombocytopenia and hemorrhage during DENV infection.

In this study, we demonstrated that DENV NS1 in the concentration, which is within the range of NS1 in dengue patients’ sera (0.01–50 μg/ml), could activate platelets and induce apoptosis in a dose-dependent manner through TLR4 signal transduction. In addition, the aggregation of NS1-activated platelets in response to subthreshold concentrations of ADP was enhanced. The adhesion of NS1-activated platelets to endothelial cells and the phagocytosis of these platelets by THP-1 cells were also increased. Because NS1 can also bind to endothelial cells and macrophage to cause their activation and cytokine release, all these effects induced by NS1 may contribute to platelet activation and thrombocytopenia during DENV infection (Fig 10)[31].

In previous studies, DENV supernatant was used to stimulate platelets, and the studies concluded that DENV infection of platelets induces platelet activation [9, 10]. However, because NS1 exists in the DENV supernatant, these results cannot exclude the possible effect of NS1 in the DENV supernatant as a cause of platelet activation. Therefore, we compared the effects of DENV supernatant on platelet activation with or without the presence of NS1. As shown in Fig 1, platelet activation induced by DENV supernatant was significantly abolished when NS1 was depleted. Furthermore, we demonstrated that the binding of NS1 to platelets was decreased when TLR4 was blocked by αTLR4 or in TLR4 knockout mice. Consistent with the binding results, blocking NS1 binding to TLR4 by antibodies or TLR4 antagonist also decreased the NS1-induced platelet activation. However, the blocking of TLR4 could not completely diminish the binding of NS1 and NS1-induced platelet activation; thus, we cannot rule out the possibility that other proteins, such as TLR2, may also be involved in NS1 binding to platelets [20, 24]. Next, we examined the properties of platelets after DENV NS1 stimulation. Like LPS, a well-known TLR4 ligand, DENV NS1 could enhance platelet aggregation in the presence of subthreshold concentrations of ADP [21]. Since NS1 could induce platelets to release ADP (S11 Fig), it is possible that the existence of these low-level ADP can only trigger platelet activation but not aggregation unless an extra-subthreshold concentration of ADP was added. In addition, previous studies have also shown that injecting mice with LPS induces profound thrombocytopenia due to the increased adherent capacity of platelets [32, 33]. In this study, we also found that the adhesion of NS1-activated platelets to endothelial cells and the phagocytosis of these platelets by THP-1 cells were also increased.

Finally, we demonstrated that DENV-induced pathogenic signs, such as thrombocytopenia, prolonged bleeding time, and local skin hemorrhage in mice, were significantly abolished in NS1-depleted DENV supernatant, but not in UV-DENV supernatant, which had no detectable viral titer but still contained NS1. It is worth mentioning that we also measured the NS1 in mouse sera 3 days after infection, and the circulating NS1 levels in UV-DENV-inoculated mice were nearly clear. This result is consistent with our previous study, which shows that the half-life of DENV NS1 is 2 days. In addition, the results showed that the concentration of circulating NS1 in NS1-depleted DENV-infected mouse sera was lower than that in DENV-infected mouse sera, indicating that circulating NS1 is important to help DENV infection/replication [13]. These results also suggested that even when NS1 was depleted from DENV virus supernatant, DENV could still infect mice and produce *de novo* NS1; however, the concentration of circulating NS1 in mouse sera was not sufficient to cause pathologic signs in mice.

Thrombocytopenia is a common feature observed in both mild and severe dengue disease and is correlated with disease severity. There are two possible mechanisms that can cause thrombocytopenia in dengue patients. One is increased platelet destruction and clearance from peripheral blood. The other is decreased production of platelets in the bone marrow [34]. The inhibition of megakaryocyte development in the bone marrow has been proposed as the key underlying mechanism causing thrombocytopenia in DENV-infected humanized mice [35]. Because the average platelet size is increased when the production of platelets is increased, a high mean platelet volume (MPV) generally indicates enhanced platelet destruction in patients [36]. In dengue patients, MPV is usually either high or normal. Therefore, excessive platelet destruction may be the main reason for thrombocytopenia in dengue patients [37, 38]. Indeed, platelets from DENV-infected patients exhibit increased activation and apoptosis profiles compared to healthy donors and patients with non-dengue febrile illness [10]. However, the mechanisms that cause platelet activation and apoptosis leading to thrombocytopenia are not fully understood. In this study, we demonstrated that platelets activated by DENV NS1 were prone to aggregation, attachment to endothelial cells, and phagocytosis by immune cells. Therefore, we suggest that NS1 is one of the key factors that may contribute to thrombocytopenia during DENV infection. While previous studies have shown little difference in the presence or absence of circulating NS1 between primary and secondary infections, secondary infections are overwhelmingly associated with more severe disease [39]. We could not exclude the possibility that other immune-related factors, such as autoantibodies against platelets, may also contribute to thrombocytopenia during DENV infection, especially during secondary infection [40–42]. Nevertheless, these results may explain why prophylactic platelet transfusion could not reduce the risk of clinical bleeding and thrombocytopenia in dengue patients because transfused platelets will be activated and cleared due to the presence of circulating NS1 and antiplatelet antibodies in dengue patients [43].

It is known that NS1 proteins from different flaviviruses reveal 50% to 80% homology; they may have conserved functions as well as unique characteristics, leading to different modes of flavivirus pathogenesis [44, 45]. Therefore, in this study, we compared the platelet-activating effect of NS1 from DENV and another flavivirus, ZIKV. The results demonstrated that DENV NS1 was much more potent than ZIKV NS1 at the same concentration in terms of inducing platelet activation, which may explain why DENV is the most prevalent flavivirus infection with clinical manifestations of thrombocytopenia.

Vascular leakage, thrombocytopenia, and cytokine storm are thought to play important roles in the complexity of dengue pathogenesis [45]. Previous studies have demonstrated that DENV NS1 can also bind to endothelial cells to induce vascular leakage and stimulate immune cells to cause a cytokine storm [45]. Thus, DENV NS1 has been considered to be a viral toxin, which is a potential corner piece in the puzzle of dengue pathogenesis [46]. In this study, we further demonstrated that NS1 may also contribute to thrombocytopenia, another characteristic clinical feature of dengue infection. It is known that activated/apoptotic platelets could further modulate monocyte inflammatory responses and endothelium hyperpermeability to cause vascular leakage during DENV infection [47, 48]. Consequently, platelet activation/apoptosis induced by DENV NS1 can not only cause thrombocytopenia but also enhance the inflammatory response and vascular leakage in dengue patients. Together, these results suggest that DENV NS1 plays a central role in the complex interplay of dengue pathogenesis. Therefore, NS1 may represent an important target that should be a focus of attention in the development of therapeutic drugs and vaccines against dengue infection [49].

## Materials and methods

### Ethics statement

All research involving healthy adult donors was approved by the National Cheng Kung University (NCKU) Hospital Institutional Review Board (IRB #A-ER-104-368). Informed written consent was obtained from volunteers following the human experimentation guidelines of the Institutional Review Board of NCKU Hospital.

All animal studies were performed in compliance with the Guide for the Care and Use of Laboratory Animals (The Chinese-Taipei Society of Laboratory Animal Sciences, 2010) and were approved by the Institutional Animal Care and Use Committee (IACUC) of NCKU under the number IACUC 105018.

### Platelet isolation

Platelets were isolated from human whole blood by following a platelet isolation protocol (Abcam). Briefly, peripheral blood was collected in vacutainer containing acid-citrate-dextrose (ACD) buffer and centrifuged at 200×*g* for 20 min to obtain platelet-rich plasma (PRP). PRP was centrifuged at 800×*g* in the presence of 100 nM prostaglandin E_1_ (PGE_1_) for another 20 min, and the platelet pellet was suspended in Tyrode’s buffer (134 mM NaCl, 2.9 mM KCl, 12 mM NaHCO_3_, 0.34 mM Na_2_HPO_4_, 1 mM MgCl_2_, 10 mM HEPES, and 3 mg/ml of BSA, at pH 7.4) containing 100 nM PGE_1_. The purity of the platelet preparations (>98% CD61^+^) was confirmed by flow cytometry.

Mouse whole blood was obtained by cardiac puncture, and mouse platelets were isolated by following a murine/mouse platelet isolation protocol. Briefly, mouse whole blood was collected in a 1-ml syringe fitted with a 25G needle containing 100 μl of 3.8% sodium citrate. After centrifuging the blood at 100×*g* for 20 min with no brake, PRP was obtained. The PRP was then centrifuged at 1000×g in the presence of 1 μM PGE1 for another 15 min, and the platelet pellet was suspended in 1 μM PGE1 containing Tyrode’s buffer.

### Cells

HUVECs (Bioresource Collection and Research Center, Taiwan) were cultured in EGM-2 (Lonza, Basel, Switzerland), and the human monocytic cell line THP-1 (Bioresource Collection and Research Center, Taiwan) was cultured in Roswell Park Memorial Institute 1640 Medium (RPMI 1640; Thermo Fisher Scientific, Waltham, MA, USA). The baby hamster kidney cell line BHK-21 and *Aedes albopictus* cell line C6/36, purchased from the Japanese Collection of Research Bioresources (JCRB Cell Bank, Japan) and the American Type Culture Collection (ATCC, Manassas, Virginia, USA), were maintained in Dulbecco’s modified Eagle’s medium (DMEM). The medium used to grow all cell types was supplemented with 10% fetal bovine serum (FBS; HyClone Laboratory, Logan, UT, USA). All cells were cultured at 37°C in a 5% CO_2_ atmosphere except for C6/36 cells, which were cultured at 28°C.

### Recombinant NS1 proteins and anti-NS1 neutralizing Abs

Recombinant DENV1-4 serotype NS1 proteins used in all experiments were produced by CTK Biotech (San Diego, CA, USA) in *Drosophila* S2 cells. Recombinant NS1 proteins were tested for endotoxin concentrations by a *Limulus* amebocyte lysate (LAL) assay using the LAL Chromogenic Endotoxin Kit (Thermo Fisher Scientific) and were shown to be endotoxin-free (<0.1 EU/ml). Recombinant ZIKV proteins used for platelet activation/apoptosis tests were produced by Sino Biological (Beijing, China) with the baculovirus-insect system. For the quantification of DENV NS1 in virus supernatant, an NS1 ELISA was carried out using paired anti-NS1 antibodies prepared in our laboratory, as described in our previous study [50]. For the quantification of ZIKV NS1 in the ZIKV supernatant, different concentrations of ZIKV recombinant NS1 protein (The Native Antigen Company, Oxfordshire, UK) were used as a standard, and an anti-ZIKV NS1 monoclonal antibody 111–5 (Leadgene, Taiwan) was used to detect NS1 by Western blotting. The image was analyzed and quantified by ImageJ (S1 Fig).

Anti-NS1 neutralizing Abs (33D2) were generated in our laboratory as previously described [22], and the 33D2 F(ab’)_2_ fragment was obtained using a Pierce^™^ F(ab')_2_ Preparation Kit (Thermo Fisher Scientific). An isotype-matched mouse IgG F(ab’)_2_ fragment (Thermo Fisher Scientific) was used to control for nonspecific binding of antibodies.

### *In vitro* platelet stimulation

A total of 1×10^7^ washed platelets (1×10^8^ per ml) were incubated with different stimulators: DENV NS1 (DENV2 NS1 was used if not described otherwise), ZIKV NS1 or LPS (L4391, Sigma-Aldrich, St. Louis, MO, USA) for the indicated times at 37°C. The activation of platelets was further confirmed by P-selectin (CD62P) surface expression and PS exposure with flow cytometry analysis.

To study the involvement of TLR4 in DENV NS1-induced platelet activation, we preincubated platelets with an anti-TLR4 antibody (GTX31675, Genetex, Irvine, CA, USA), the TLR4 antagonist LPS-Rs from the photosynthetic bacterium *Rhodobacter sphaeroides* (InvivoGen, San Diego, CA, USA) or the TLR4 signaling inhibitor TAK242 (Sigma-Aldrich) for 30 min and then treated them with DENV NS1 for 1 h. To exclude the possibility of LPS-induced platelet activation, we used the LPS inhibitor polymyxin B (PMB) (Sigma-Aldrich) (10 μg/ml). The inhibitory functions of these inhibitors and anti-TLR4 antibody were confirmed by inhibiting LPS-induced MIF secretion in PMA-activated THP-1 cells. Briefly, PMA-activated THP-1 cells were stimulated with LPS (1 μg/ml) for 24 h. The concentration of human MIF in the cell culture medium was measured using human MIF ELISA kits (R&D Systems, Minneapolis, MN, USA) following the manufacturers’ instructions.

### Flow cytometry

To confirm the purity of isolated platelets, the surface expression of the platelet marker CD61 was determined using a specific antibody. Briefly, 1×10^7^ washed platelets were incubated with anti-CD61 monoclonal antibody (GTX61848, Genetex, Irvine, CA, USA) in 0.01% NaN_3_ and 1% BSA containing Tyrode’s buffer at 4°C for 1 h, followed by Alexa 594-conjugated goat anti-rabbit IgG secondary antibody (1:500; Thermo Fisher Scientific) incubation for another 30 min.

P-selectin (CD62P) surface expression was determined using FITC-conjugated anti-human CD62P (BD Bioscience, San Diego, CA). Platelets (1×10^7^) were incubated with FITC-conjugated anti-human CD62P (1:10) or FITC-conjugated isotype-matched antibodies (BD Bioscience). PS exposure was detected by staining with a fluorescent conjugate of Annexin V, a protein that has a high affinity for PS, using an Alexa Fluor 594 Annexin V/Dead Cell Apoptosis Kit (Thermo Fisher Scientific) according to the manufacturer’s instructions.

To quantify the binding of NS1 on platelet surfaces, a quantity of 1×10^7^ washed platelets was preincubated with or without an anti-TLR4 antibody (5 μg/ml, Genetex) in 0.1% NaN_3_ and 1 μM PGE1 containing Tyrode’s buffer at 4°C for 1 h. NS1 (5 μg/ml) and FITC-conjugated anti-NS1 monoclonal antibodies (33D2-FITC) were added and then incubated at 4°C for another 3 h. The percent fluorescence signal on the platelet surface was analyzed by FACSCalibur flow cytometry. Data were analyzed using FlowJo software (Tree Star, Ashland, OR, USA).

### Light transmission aggregometry

Human whole blood was collected in vacutainers containing sodium citrate buffer and centrifuged at 1000 rpm for 15 min to obtain PRP. Platelet-poor plasma (PPP), used as a blank control, was obtained by centrifuging at 3000 rpm for 15 min. A total of 450 μl of PRP or PPP was pipetted into an aggregometry cuvette, which was preheated to 37°C for 5 min. The light transmission was measured in a Chrono-log aggregometer (Helena Laboratories, Beaumont, Texas, USA) for 5 min after ADP (10 μM) or NS1 was added.

For the platelet aggregation enhancement assay, PRP was preincubated with BSA or NS1 (with or without the F(ab’)_2_ antibody fragment) at 37°C for 1 h. To investigate whether the NS1-induced enhancement of platelet aggregation was mediated by ADP receptors, PRP was preincubated with BSA or NS1 (with or without ADP receptors inhibitors, BMS-646786 (BM0020, Sigma-Aldrich) or Clopidogrel hydrogen sulfate (SML0004, Sigma-Aldrich)) at 37°C for 1 h. The light transmission was measured for 5 min after low-dose ADP (2.5 μM) was added.

### Indirect immunofluorescence assay

To visualize the binding of NS1 on platelet surfaces, 2×10^6^ washed platelets were plated on 0.01% poly-L-lysine-coated coverslips in 24-well plates and treated with BSA or NS1 (1 μg/ml) at 4°C for 1 h. Subsequently, platelets were fixed with 1% paraformaldehyde with no further permeabilization. After blocking with SuperBlock^™^ (PBS) Blocking Buffer (Thermo Fisher Scientific) for 1 h, platelets were incubated overnight at 4°C with anti-NS1 monoclonal antibodies (33D2) and anti-CD61 monoclonal antibody (Genetex). After being washed with PBS, platelets were incubated with Alexa 488-conjugated goat anti-mouse IgG secondary antibody and Alexa 594-conjugated goat anti-rabbit IgG secondary antibody (1:500; Thermo Fisher Scientific) for 1 h. The coverslips were mounted with VECTASHIELD Antifade Mounting Medium (Vector Laboratories, Burlingame, CA, USA). The images were acquired using a confocal microscope (Olympus FluoView FV1000).

### Western blotting

The protein expression levels of TLR4, caspase-3 and β actin in human-isolated platelets were detected using an anti-TLR4 antibody (GTX31675, Genetex), an anti-caspase-3 antibody (IMG-144A, Novus Biologicals, Centennial, CO, USA) and an anti-β actin antibody (Arigo, Taiwan). The amount of NS1 in concentrated viral supernatant was detected using an anti-DENV NS1 antibody (33D2). After overnight incubation, the membranes were incubated with a 1:10,000 dilution of horseradish peroxidase (HRP)-conjugated anti-mouse or anti-rabbit immunoglobulin antibody (Leadgene). The bound HRP-conjugated antibodies were detected using WesternBright ECL (Advansta, San Jose, CA, USA).

### Enzyme-linked immunosorbent assay (ELISA)

To examine the direct interaction between DENV NS1 and candidate receptors, 50 μl of TLR4 (purified from baculovirus-system, Sino Biological), TLR2 (purified from baculovirus-system, Sino Biological), His-taq protein or BSA (5 μg/ml) in PBS (pH 7.3) was coated onto 96-well ELISA plates at 4 °C overnight. After blocking at 37 °C for 1 h with 1% BSA in PBS, DENV NS1 was serially diluted (from 1 μg/ml) in Diluent buffer (LG1 buffer, Leadgene) and incubated in wells at 37 °C for 1 h. After washing three times with PBST (PBS contained 0.01% Tween 20), the bound NS1 was detected with biotin-labeled anti-NS1 antibodies (1 μg/ml). Next, HRP-labeled streptavidin solution (1:200) (R&D) was added to wells at 37°C for 20 min. After washing three times with PBST, color development with TMB was performed. The absorbance was read following the addition of stop solution (2N H_2_SO_4_) at OD_450_ nm by a VersaMax microplate reader.

### Platelet adhesion assay

Platelets with or without NS1 stimulation under different conditions for 1 h were washed twice with Tyrode’s buffer before incubation with HUVECs to exclude the effect of excessive DENV NS1. HUVECs, which were grown to confluence on coverslips in 24-well plates, were cultured with 1×10^7^ platelets per well in Tyrode’s buffer for 1 h at 37°C. The cells on the coverslips were washed twice with PBS/2% FBS to remove nonadhered platelets, fixed with 1% paraformaldehyde and permeabilized with 0.1% Triton X-100. To assay adhered platelets, we incubated the cells overnight at 4°C with an anti-CD61 monoclonal antibody, followed by Alexa 488-conjugated goat anti-rabbit IgG secondary antibody (1:500 diluted; Thermo Fisher Scientific) and Hoechst 33342 (1:3,000 diluted; Thermo Fisher Scientific). After the specimens were mounted with VECTASHIELD Antifade Mounting Medium, the images were acquired using a confocal microscope (Olympus FluoView FV1000).

### Transwell permeability assay

HUVECs (1× 10^5^) were grown on the upper chambers of Transwell plates (0.4 μm; Corning, The Netherlands) until a monolayer was formed. The HUVECs monolayer was coincubated with BSA or NS1-treated washed platelets (1×10^7^, 200 μl) along with Tyrode’s buffer. After the indicated time point, the upper chambers were reconstituted with 300 μl serum-free media, which contained 3 μl of streptavidin-horseradish peroxidase (HRP) (R&D Systems). Fifty microliters of medium in the lower chamber was collected 10 min after adding streptavidin-HRP and was assayed for HRP activity by adding 100 μl of 3,3',5,5'-tetramethylbenzidine (TMB) substrate (R&D Systems). The color development was detected by a VersaMax microplate reader (Molecular Devices, Sunnyvale, CA) at 450 nm.

### Platelet phagocytic assay

For the platelet phagocytic assay, THP-1 cells (2×10^6^ per ml) were activated with PMA (10 ng/ml) (Sigma-Aldrich) and plated on coverslips in 24-well plates. After 48 h, the PMA-containing medium was removed, and 1×10^7^ washed platelets, which were subjected to different conditions of NS1 stimulation, were added to the phagocyte in Tyrode’s buffer for 4 h at 37°C. The phagocyte monolayer was washed twice with PBS/2% FBS, followed by fixation and permeabilization. To test whether NS1-activated platelets could be engulfed by non-PMA-activated THP-1 cells, 5×10^5^ THP-1 cells were coincubated with 1×10^7^ washed platelets in Tyrode’s buffer at 37°C. After 4 h, the mixed cells were concentrated and formed a monolayer on the slide by using the cytospin method (800 rpm for 5 min with no brake), followed by fixation and permeabilization. To assay adhered/phagocytic platelets, we incubated the phagocyte monolayer overnight with anti-CD61 monoclonal antibody and anti-CD14 monoclonal antibody (sc-1182, Santa Cruz, Dallas, Texas, USA) followed by Alexa 488-conjugated goat anti-rabbit IgG secondary antibody (1:500 diluted; Thermo Fisher Scientific) and Alexa 594-conjugated goat anti-mouse IgG secondary antibody (1:500 diluted; Thermo Fisher Scientific). After the specimens were mounted with VECTASHIELD Antifade Mounting Medium, the images were acquired using a confocal microscope (Olympus FluoView FV1000).

### THP-1 activation assay

To test whether NS1-activated platelets could trigger THP-1 cell activation, 5×10^5^ THP-1 cells were coincubated with 1×10^7^ washed platelets in 12-well plates with Tyrode’s buffer at 37°C for indicated time, and THP-1 cells were treated with 10 ng/ml PMA for 48 h for the positive control group. The cell adherence and activation-related mRNA expression levels were determined according to a previous study [29]. The mRNA expression levels of monocyte chemoattractant protein-1 (MCP-1/CCL2) and glyceraldehyde 3-phosphate dehydrogenase (GAPDH), a house-keeping gene, in THP-1 after incubation with washed platelets were determined by semiquantitative RT-PCR using a PyroRTase kit and 2X Taq PCR Master Mix (Leadgene). The cell adherence after treatment was measured by counting the suspended cells in the supernatants.

### HUVECs, THP-1 cells and platelet coculture model

A total of 5×10^5^ PMA-activated THP-1 cells and 1×10^7^ platelets were added to confluent HUVECs on coverslips in 24-well plates, followed by treatment with BSA or NS1 (with or without the F(ab’)_2_ antibody fragment) at 37°C for 1 h. The supernatants from indicated experimental groups were collected, and lactate dehydrogenase (LDH) secretion was measured by LDH release assay (Promega, Madison, WI, USA). The cells were washed twice with PBS/2% FBS, fixed with 1% paraformaldehyde and permeabilized with 0.1% Triton X-100. After being blocked, the cells were incubated overnight with anti-CD61 monoclonal antibody (Genetex) and anti-CD14 monoclonal antibody (Santa Cruz) followed by Alexa 488-conjugated goat anti-rabbit IgG secondary antibody, Alexa 594-conjugated goat anti-mouse IgG secondary antibody (1:500; Thermo Fisher Scientific), and Hoechst 33342 (Invitrogen, Carlsbad, CA, USA) (1:3,000 diluted) for 1 h. After the specimens were mounted with VECTASHIELD Antifade Mounting Medium (Vector Laboratories, Burlingame, CA, USA), the images were acquired using a confocal microscope (Olympus FluoView FV1000).

### Viruses

DENV serotype 2 local Taiwan strain 454009 A and ZIKV Asian strain PRVABC were propagated in C6/36 cells as previously described [51]. To prepare high titers of DENV, we concentrated DENV-containing culture supernatants with Macrosep Advance Centrifugal Devices (molecular weight cutoff of 30 kDa; Pall Corp., Port Washington, NY, USA) at 6000×*g* at 4°C and stored below −70°C until use. UV-inactivated DENV (UV-DENV) was obtained by treatment with UV 100 mJ/cm^2^ for 100 s in a UV-crosslinker (VILBER, France). To deplete NS1, we incubated DENV-containing culture supernatant with mouse anti-dengue NS1 1D33-agarose (Leadgene) at 4°C for 1 h, followed by centrifugation at 3000×*g* at 4°C to obtain NS1-depleted DENV supernatant. The virus titer and NS1 concentration in each condition were analyzed by fluorescent focus assay and NS1 ELISA after the process.

### Mouse model

C3H/HeN and C57BL/6J mice aged 6 to 8 weeks old, obtained from the National Laboratory Animal Center, were used in a DENV-induced hemorrhage murine model [30]. The *Tlr4*^*-/-*^ mice, aged 6–8 weeks old, were kindly provided by Prof. Pei-Jane Tsai (NCKU) and maintained on a C57BL/6J genetic background. Briefly, mice were intradermally inoculated with serotype 2 of DENV at four sites on the upper back (50 μl/site) after being anesthetized with Zoletil 50 (Virbac, France). In some mice, mock C6/36 culture supernatant, an equivalent titer of UV-DENV, or an equivalent titer of NS1-depleted DENV, or DENV (2×10^8^ PFU/mouse) were inoculated. To observe hemorrhage development, we sacrificed the mice on day 3 after inoculation and exposed the subcutaneous tissues of the back.

### Platelet count

Mouse blood was collected by orbital sinus sampling on day 3 after inoculation and immediately mixed with 0.1% EDTA. Platelets were counted using a Scil-Vet Animal Care Vet Focus 5 Hematology Analyzer (Mindray, China) through a service provided by the animal center of NCKU.

### Bleeding time

The bleeding time in mice was measured by cutting off 3–5 mm from the tips of the tails. The blood was dropped onto filter paper every 30 s until the diameter of the blood droplet was smaller than 0.5 mm, and the duration of bleeding was recorded.

### Statistical analysis

The *in vitro* and *in vivo* data are expressed as the means ± standard deviations (SD) from more than three independent experiments. Student’s t-test was used to analyze the significance of differences between the test and control groups. One-way ANOVA with a Kruskal-Wallis comparison test was used to analyze the significance of differences between multiple groups. All data were analyzed by GraphPad Prism 5 software. P values <0.05 were considered statistically significant.

## Supporting information

S1 Fig Quantification of NS1 in DENV and ZIKV viral supernatant. **(A)** After 10-fold concentration, the viral titers of DENV and ZIKV were determined by fluorescent focus assay. The NS1 concentration in the DENV concentrated supernatant was determined by an NS1 enzyme-linked immunosorbent assay, as described in a previous study [50]. **(B)(C)** The NS1 concentration of the ZIKV supernatant was analyzed and quantified by Western blotting using ZIKV recombinant NS1 (The Native Antigen Company) as a standard, as described in the Materials and Methods.
(DOCX)

S2 Fig Representative plots for flow cytometry analysis of Fig 1. Human-isolated platelets were stained with anti-P-selectin (FITC) or Annexin V (PE). The percent fluorescence of P-selectin surface expression on platelets and annexin V binding to platelets were analyzed by FACSCalibur flow cytometry. Data analysis was performed with FlowJo software (FlowJo, LLC).
(DOCX)

S3 Fig DENV NS1, but not ZIKV NS1, induces platelet activation and apoptosis. Platelets were treated with BSA, DENV NS1 or ZIKV NS1 recombinant proteins (10 μg/ml) for 1 h and stained with anti-P-selectin (FITC) or Annexin V (PE) (n = 3). **(A)** The percent fluorescence of P-selectin surface expression on platelets and **(B)** annexin V binding to platelets were analyzed by FACSCalibur flow cytometry. The representative FACS plots were constructed using FLOWJO software. *P<0.05; Kruskal-Wallis ANOVA (panels A and B).
(DOCX)

S4 Fig Representative plots for flow cytometry analysis of Fig 2. The percent fluorescence of P-selectin surface expression on platelets was analyzed by FACSCalibur flow cytometry, and the data analysis was performed with FlowJo software (FlowJo, LLC).
(DOCX)

S5 Fig Representative plots for flow cytometry analysis of Fig 3. The percent fluorescence of annexin V binding to platelets was analyzed by FACSCalibur flow cytometry, and the data analysis was performed with FlowJo software (FlowJo, LLC).
(DOCX)

S6 Fig DENV NS1 induces caspase-3 activation in platelets. Human-isolated platelets were treated with BSA, DENV NS1 (10 μg/ml) or human thrombin (0.1 U/ml) for the indicated time. The caspase-3 activation was analyzed by Western blotting (50 μg protein/lane). The relative values (cleaved caspase-3/β actin) are shown in the figure.
(DOCX)

S7 Fig DENV NS1 binds to platelet surfaces. The binding of NS1 on platelet surfaces was determined by both **(A)** indirect immunofluorescence assay (IFA) and **(B)** flow cytometry. For IFA, platelets were plated on 0.01% poly-L-lysine-coated coverslips and incubated with BSA or DENV NS1 (10 μg/ml) in Tyrode’s buffer (containing 0.01% NaN3 and 1 μM PGE1) for 1 h at 4°C before fixation. Platelets were stained with anti-CD61 and mouse anti-NS1 mAb (33D2), followed by anti-mouse Alexa 488-conjugated antibody and anti-rabbit Alexa 594-conjugated antibody. For flow cytometry, platelets were incubated with BSA, DENV NS1 (10 μg/ml), FITC-conjugated anti-NS1 monoclonal antibodies (33D2-FITC) or FITC-conjugated control mouse IgG (cmIgG-FITC) in Tyrode’s buffer (containing 0.01% NaN3 and 1 μM PGE1) for 3 h at 4°C. The percent fluorescence of NS1 binding on platelets was analyzed by FACSCalibur flow cytometry, and the data analysis was performed with FlowJo software (FlowJo, LLC).
(DOCX)

S8 Fig Representative plots for flow cytometry analysis of Fig 4 and the inhibitory effects of inhibitors. **(A-F)** The percent fluorescence of NS1 binding and P-selectin surface expression on platelets was analyzed by FACSCalibur flow cytometry. Data analysis was performed with FlowJo software (FlowJo, LLC). (G) PMA-activated THP-1 cells were pretreated with TAK242 (10 μM), LPS-Rs (10 μg/ml), αTLR4 (5 μg/ml) or control rabbit IgG (5 μg/ml) for 30 min (or cotreated with PMB (10 μg/ml)), followed by LPS (1 μg/ml) stimulation for 24 h (n = 3 per group). Cell supernatants were collected, and the concentrations of MIF in the cell supernatants were determined by human MIF ELISA kits.
(DOCX)

S9 Fig DENV NS1 could interact with both TLR4 and TLR2. The binding of DENV NS1 to TLR4, TLR2, His-taq protein or BSA (5 μg/ml) was analyzed by ELISA, as described in the Methods.
(DOCX)

S10 Fig LPS at a high dose could induce platelet activation. Human-isolated platelets were stimulated with different concentrations of LPS or DENV NS1 (10 μg/ml) for 1 h (n = 5). The percent fluorescence of P-selectin surface expression on platelets was analyzed by FACSCalibur flow cytometry, and the data analysis was performed with FlowJo software (FlowJo, LLC). *P<0.05, **P<0.01; Kruskal-Wallis ANOVA (panel B).
(DOCX)

S11 Fig DENV NS1 induces platelets to secrete ADP. **(A)(B)** Human-isolated platelets were stimulated with BSA, DENV NS1 (10 μg/ml) or thrombin (0.1 U/ml) for the indicated time, and the ADP in the supernatant was measured by ADP assay kit (ab83359, Abcam, Cambridge, UK). **(C)** PRP was treated with BSA or DENV NS1 (10 μg/ml) (cotreated with or without 1 μM BPTU or 1 μM Clopidogrel (both are ADP receptor inhibitors, Sigma-Aldrich) for 1 h and stimulated with ADP (2.5 μM). The light transmission of PRP was measured in a Chrono-log aggregometer.
(DOCX)

S12 Fig DENV NS1-activated platelets trigger endothelial hyperpermeability. HUVEC monolayers were coincubated with washed NS1-activated platelets for the indicated time, and the relative endothelial permeability was assessed by a Transwell permeability assay, as described in the Methods.
(DOCX)

S13 Fig THP-1 activation and phagocytosis induced by coculture with DENV NS1-activated platelets. **(A)** For the phagocytosis assay, washed NS1-activated platelets (1x10^7^) were cultured with THP-1 cells for 4 h and were concentrated onto the slide using cytospin. After fixation and permeabilization, the cells were stained with CD61 (green, a marker of platelets) and CD14 (red, a marker of monocytes/macrophages) and examined by confocal microscopy. The yellow dots represent the engulfed platelets. **(B)(C)** THP-1 cells (5x10^5^) were incubated with washed NS1-activated platelets (1x10^7^) for the indicated time (positive control: PMA-treated THP-1 cells for 48 h). After incubation, MCP-1 mRNA expression and adherence of THP-1 cells to plates were determined.
(DOCX)

S14 Fig Viral titer and NS1 concentrations in different conditions of DENV supernatant. The virus titer was determined by fluorescent focus assay, and the NS1 concentration was analyzed by **(A)** NS1 enzyme-linked immunosorbent assay and **(B)** Western blotting after the experiment.
(DOCX)

S15 Fig Classification of skin hemorrhage into four grades according to hemorrhage severity. (DOCX)

S16 Fig NS1 secretion in sera of DENV 2-infected mice. C3H/HeN mice (n = 5) were inoculated with DENV (2x10^8^ PFU/mouse), UV-inactivated DENV or NS1-depleted DENV on the upper back. Mouse sera were collected at 3 days after inoculation, and the NS1 secretion level in mice was analyzed by NS1 quantitative ELISA. *P<0.05, **P<0.01; Kruskal-Wallis ANOVA.
(DOCX)
