# Supplementary material for: Dengue virus nonstructural protein 1 activates platelets via Toll-like receptor 4, leading to thrombocytopenia and hemorrhage
Source: PLoS Pathog. 2019 Apr 22;15(4):e1007625. doi: 10.1371/journal.ppat.1007625 (PMC6497319; doi:10.1371/journal.ppat.1007625)
Supplement: S1 Fig — (A) After 10-fold concentration, the viral titers of DENV and ZIKV were determined by fluorescent focus assay. The NS1 concentration in the DENV concentrated supernatant was determined by an NS1 enzyme-linked immunosorbent assay, as described in a previous study [50]. (B)(C) The NS1 concentration of the ZIKV supernatant was analyzed and quantified by Western blotting using ZIKV recombinant NS1 (The Native Antigen Company) as a standard, as described in the Materials and Methods. (DOCX) [file ppat.1007625.s001.docx]

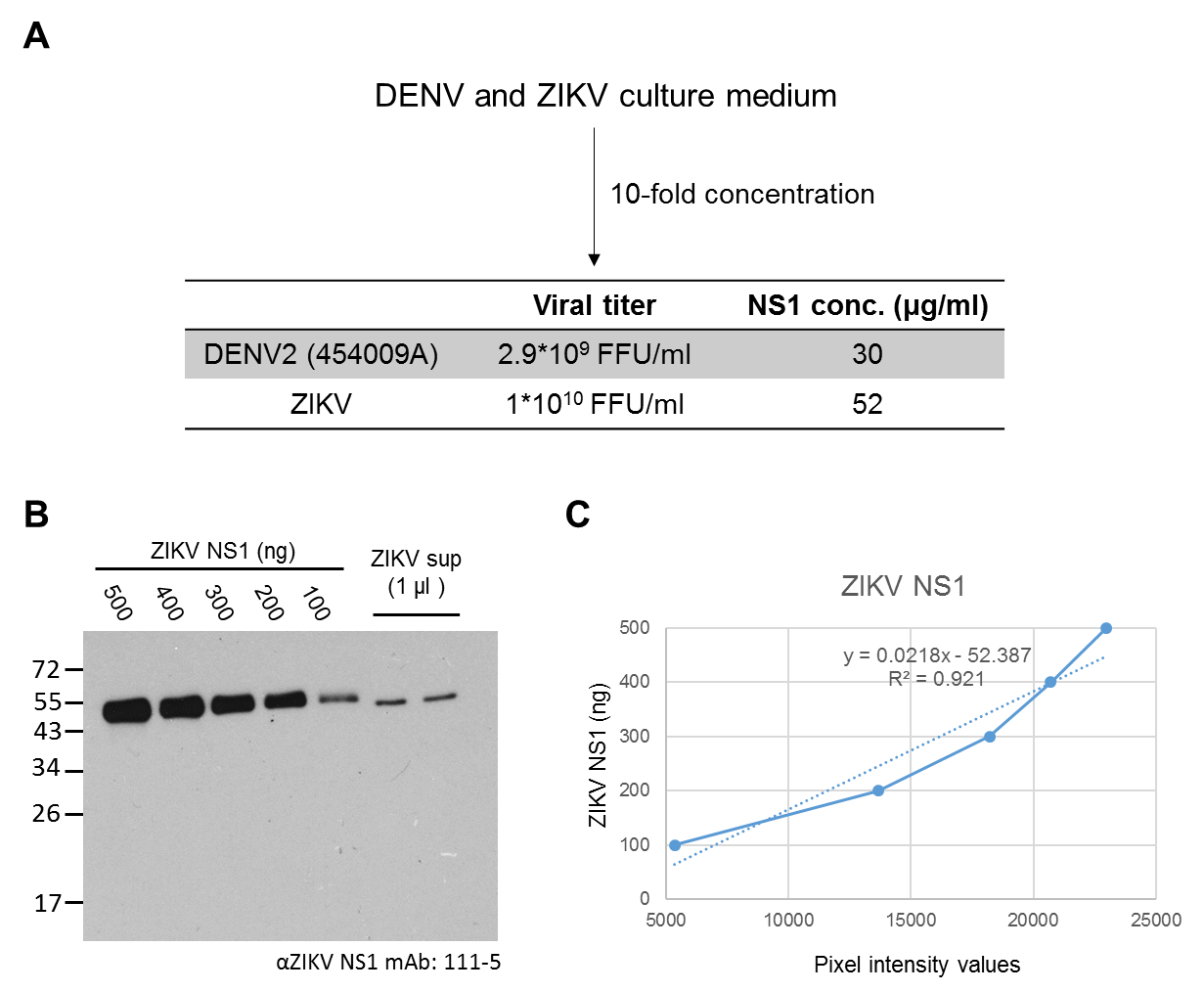


**S1 Fig. Quantification of NS1 in DENV and ZIKV viral supernatant. (A)** After 10-fold concentration, the viral titers of DENV and ZIKV were determined by fluorescent focus assay. The NS1 concentration in the DENV concentrated supernatant was determined by an NS1 enzyme-linked immunosorbent assay, as described in a previous study [50]. **(B)(C)** The NS1 concentration of the ZIKV supernatant was analyzed and quantified by Western blotting using ZIKV recombinant NS1 (The Native Antigen Company) as a standard, as described in the Materials and Methods.
