# Supplementary material for: Dengue virus nonstructural protein 1 activates platelets via Toll-like receptor 4, leading to thrombocytopenia and hemorrhage
Source: PLoS Pathog. 2019 Apr 22;15(4):e1007625. doi: 10.1371/journal.ppat.1007625 (PMC6497319; doi:10.1371/journal.ppat.1007625)
Supplement: S2 Fig — Human-isolated platelets were stained with anti-P-selectin (FITC) or Annexin V (PE). The percent fluorescence of P-selectin surface expression on platelets and annexin V binding to platelets were analyzed by FACSCalibur flow cytometry. Data analysis was performed with FlowJo software (FlowJo, LLC). (DOCX) [file ppat.1007625.s002.docx]

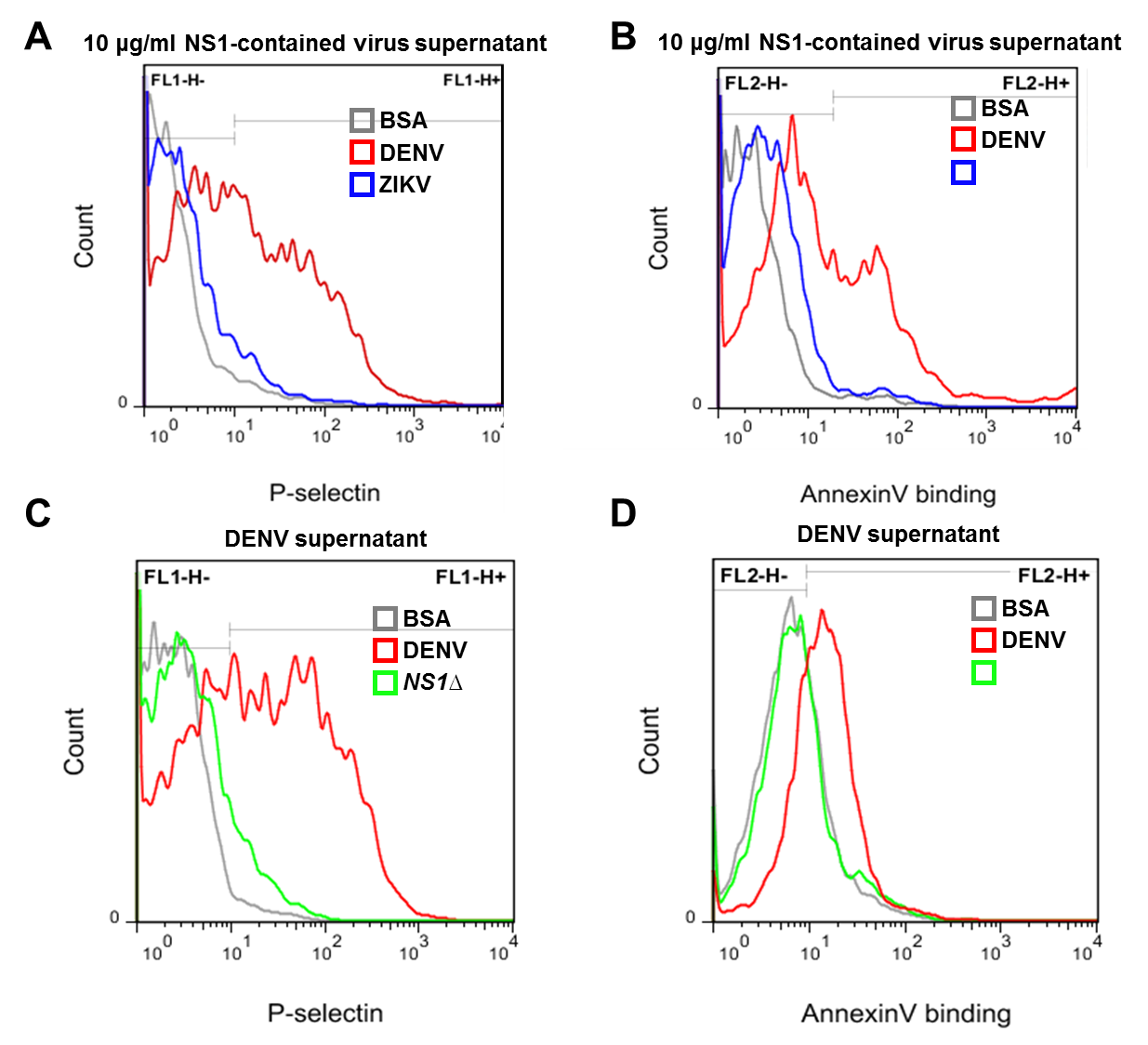


**S2 Fig.** **Representative plots for flow cytometry analysis of Figure 1.** Human-isolated platelets were stained with anti-P-selectin (FITC) or Annexin V (PE). The percent fluorescence of P-selectin surface expression on platelets and annexin V binding to platelets were analyzed by FACSCalibur flow cytometry. Data analysis was performed with FlowJo software (FlowJo, LLC).
