# Supplementary material for: Dengue virus nonstructural protein 1 activates platelets via Toll-like receptor 4, leading to thrombocytopenia and hemorrhage
Source: PLoS Pathog. 2019 Apr 22;15(4):e1007625. doi: 10.1371/journal.ppat.1007625 (PMC6497319; doi:10.1371/journal.ppat.1007625)
Supplement: S3 Fig — Platelets were treated with BSA, DENV NS1 or ZIKV NS1 recombinant proteins (10 μg/ml) for 1 h and stained with anti-P-selectin (FITC) or Annexin V (PE) (n = 3). (A) The percent fluorescence of P-selectin surface expression on platelets and (B) annexin V binding to platelets were analyzed by FACSCalibur flow cytometry. The representative FACS plots were constructed using FLOWJO software. *P<0.05; Kruskal-Wallis ANOVA (panels A and B). (DOCX) [file ppat.1007625.s003.docx]

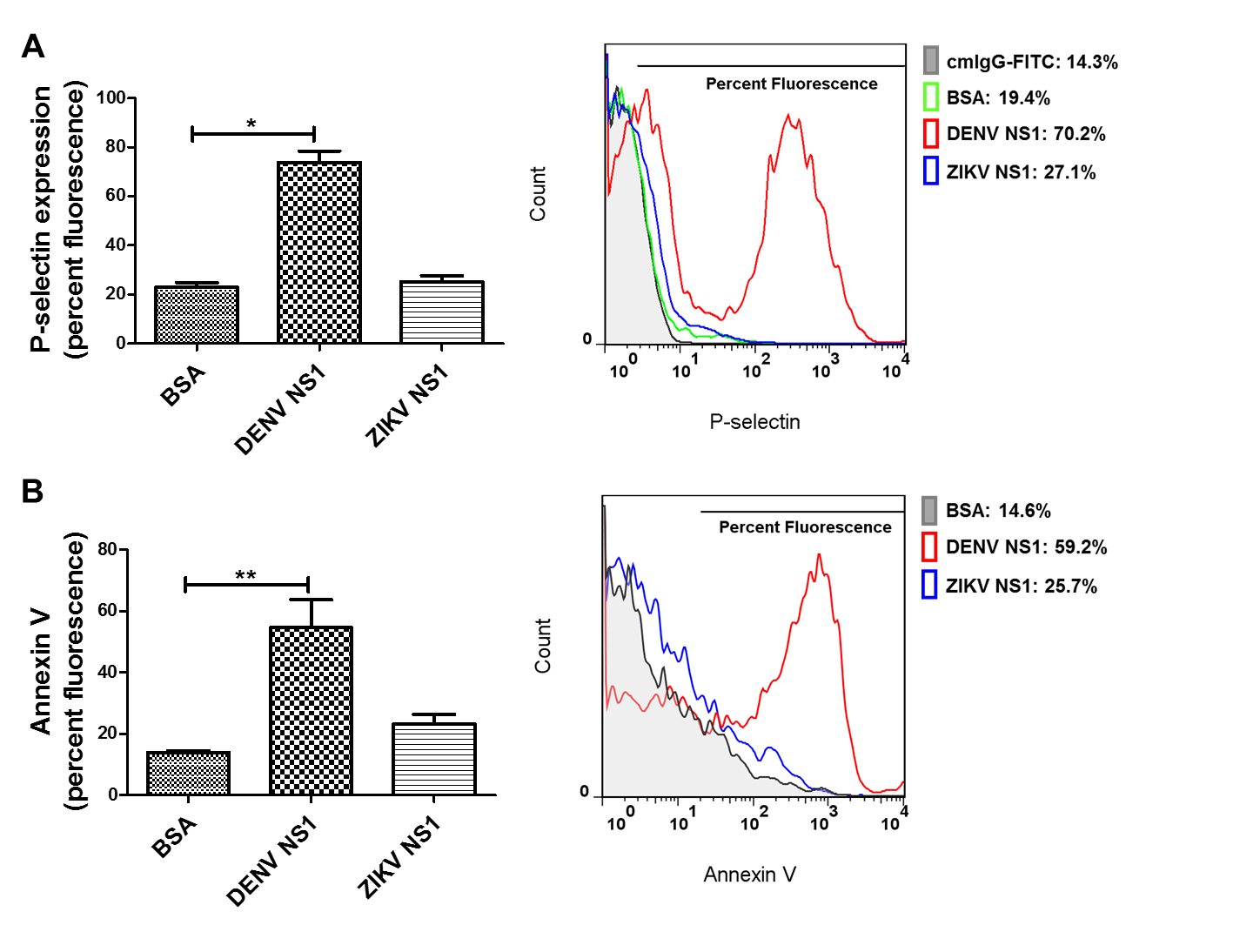


**S3 Fig. DENV NS1, but not ZIKV NS1, induces platelet activation and apoptosis.** Platelets were treated with BSA, DENV NS1 or ZIKV NS1 recombinant proteins (10 μg/ml) for 1 h and stained with anti-P-selectin (FITC) or Annexin V (PE) (n=3). **(A)** The percent fluorescence of P-selectin surface expression on platelets and **(B)** annexin V binding to platelets were analyzed by FACSCalibur flow cytometry. The representative FACS plots were constructed using FLOWJO software. *P<0.05; Kruskal-Wallis ANOVA (panels A and B).
