# Supplementary material for: Dengue virus nonstructural protein 1 activates platelets via Toll-like receptor 4, leading to thrombocytopenia and hemorrhage
Source: PLoS Pathog. 2019 Apr 22;15(4):e1007625. doi: 10.1371/journal.ppat.1007625 (PMC6497319; doi:10.1371/journal.ppat.1007625)
Supplement: S5 Fig — The percent fluorescence of annexin V binding to platelets was analyzed by FACSCalibur flow cytometry, and the data analysis was performed with FlowJo software (FlowJo, LLC). (DOCX) [file ppat.1007625.s005.docx]

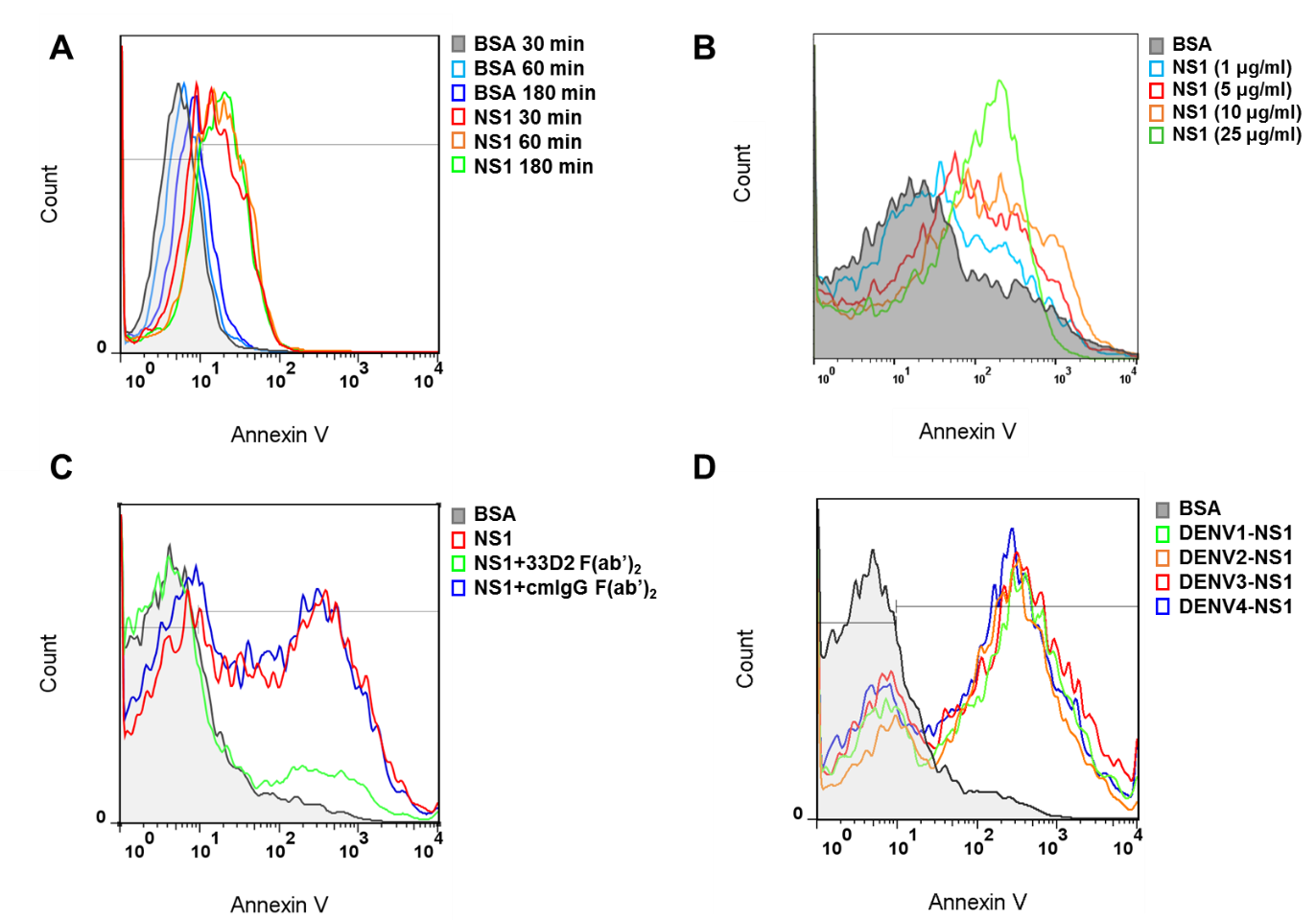


**S5 Fig. Representative plots for flow cytometry analysis of Figure 3.** The percent fluorescence of annexin V binding to platelets was analyzed by FACSCalibur flow cytometry, and the data analysis was performed with FlowJo software (FlowJo, LLC).
