# Supplementary material for: Dengue virus nonstructural protein 1 activates platelets via Toll-like receptor 4, leading to thrombocytopenia and hemorrhage
Source: PLoS Pathog. 2019 Apr 22;15(4):e1007625. doi: 10.1371/journal.ppat.1007625 (PMC6497319; doi:10.1371/journal.ppat.1007625)
Supplement: S6 Fig — Human-isolated platelets were treated with BSA, DENV NS1 (10 μg/ml) or human thrombin (0.1 U/ml) for the indicated time. The caspase-3 activation was analyzed by Western blotting (50 μg protein/lane). The relative values (cleaved caspase-3/β actin) are shown in the figure. (DOCX) [file ppat.1007625.s006.docx]

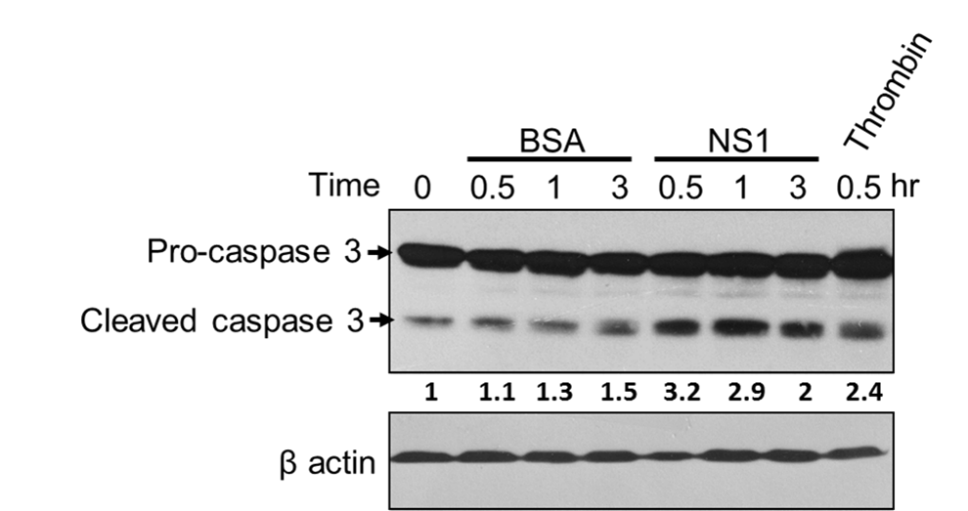


**S6 Fig. DENV NS1 induces caspase-3 activation in platelets.** Human-isolated platelets were treated with BSA, DENV NS1 (10 μg/ml) or human thrombin (0.1 U/ml) for the indicated time. The caspase-3 activation was analyzed by Western blotting (50 μg protein/lane). The relative values (cleaved caspase-3/β actin) are shown in the figure.
