# Supplementary material for: Dengue virus nonstructural protein 1 activates platelets via Toll-like receptor 4, leading to thrombocytopenia and hemorrhage
Source: PLoS Pathog. 2019 Apr 22;15(4):e1007625. doi: 10.1371/journal.ppat.1007625 (PMC6497319; doi:10.1371/journal.ppat.1007625)
Supplement: S7 Fig — The binding of NS1 on platelet surfaces was determined by both (A) indirect immunofluorescence assay (IFA) and (B) flow cytometry. For IFA, platelets were plated on 0.01% poly-L-lysine-coated coverslips and incubated with BSA or DENV NS1 (10 μg/ml) in Tyrode’s buffer (containing 0.01% NaN3 and 1 μM PGE1) for 1 h at 4°C before fixation. Platelets were stained with anti-CD61 and mouse anti-NS1 mAb (33D2), followed by anti-mouse Alexa 488-conjugated antibody and anti-rabbit Alexa 594-conjugated antibody. For flow cytometry, platelets were incubated with BSA, DENV NS1 (10 μg/ml), FITC-conjugated anti-NS1 monoclonal antibodies (33D2-FITC) or FITC-conjugated control mouse IgG (cmIgG-FITC) in Tyrode’s buffer (containing 0.01% NaN3 and 1 μM PGE1) for 3 h at 4°C. The percent fluorescence of NS1 binding on platelets was analyzed by FACSCalibur flow cytometry, and the data analysis was performed with FlowJo software (FlowJo, LLC). (DOCX) [file ppat.1007625.s007.docx]

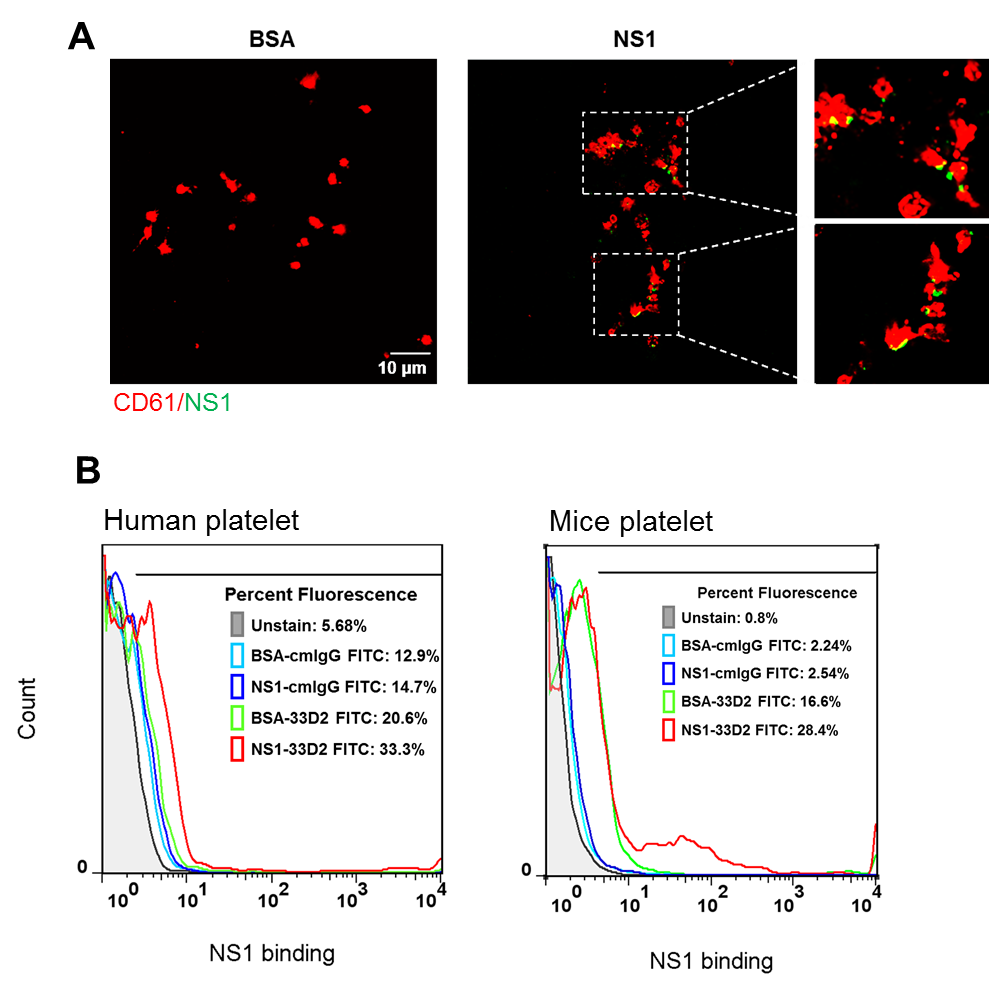


**S7 Fig. DENV NS1 binds to platelet surfaces.** The binding of NS1 on platelet surfaces was determined by both **(A)** indirect immunofluorescence assay (IFA) and **(B)** flow cytometry. For IFA, platelets were plated on 0.01% poly-L-lysine-coated coverslips and incubated with BSA or DENV NS1 (10 μg/ml) in Tyrode’s buffer (containing 0.01% NaN3 and 1 µM PGE1) for 1 h at 4ºC before fixation. Platelets were stained with anti-CD61 and mouse anti-NS1 mAb (33D2), followed by anti-mouse Alexa 488-conjugated antibody and anti-rabbit Alexa 594-conjugated antibody. For flow cytometry, platelets were incubated with BSA, DENV NS1 (10 μg/ml), FITC-conjugated anti-NS1 monoclonal antibodies (33D2-FITC) or FITC-conjugated control mouse IgG (cmIgG-FITC) in Tyrode’s buffer (containing 0.01% NaN3 and 1 μM PGE1) for 3 h at 4℃. The percent fluorescence of NS1 binding on platelets was analyzed by FACSCalibur flow cytometry, and the data analysis was performed with FlowJo software (FlowJo, LLC).
