# Supplementary material for: Dengue virus nonstructural protein 1 activates platelets via Toll-like receptor 4, leading to thrombocytopenia and hemorrhage
Source: PLoS Pathog. 2019 Apr 22;15(4):e1007625. doi: 10.1371/journal.ppat.1007625 (PMC6497319; doi:10.1371/journal.ppat.1007625)
Supplement: S8 Fig — (A-F) The percent fluorescence of NS1 binding and P-selectin surface expression on platelets was analyzed by FACSCalibur flow cytometry. Data analysis was performed with FlowJo software (FlowJo, LLC). (G) PMA-activated THP-1 cells were pretreated with TAK242 (10 μM), LPS-Rs (10 μg/ml), αTLR4 (5 μg/ml) or control rabbit IgG (5 μg/ml) for 30 min (or cotreated with PMB (10 μg/ml)), followed by LPS (1 μg/ml) stimulation for 24 h (n = 3 per group). Cell supernatants were collected, and the concentrations of MIF in the cell supernatants were determined by human MIF ELISA kits. (DOCX) [file ppat.1007625.s008.docx]

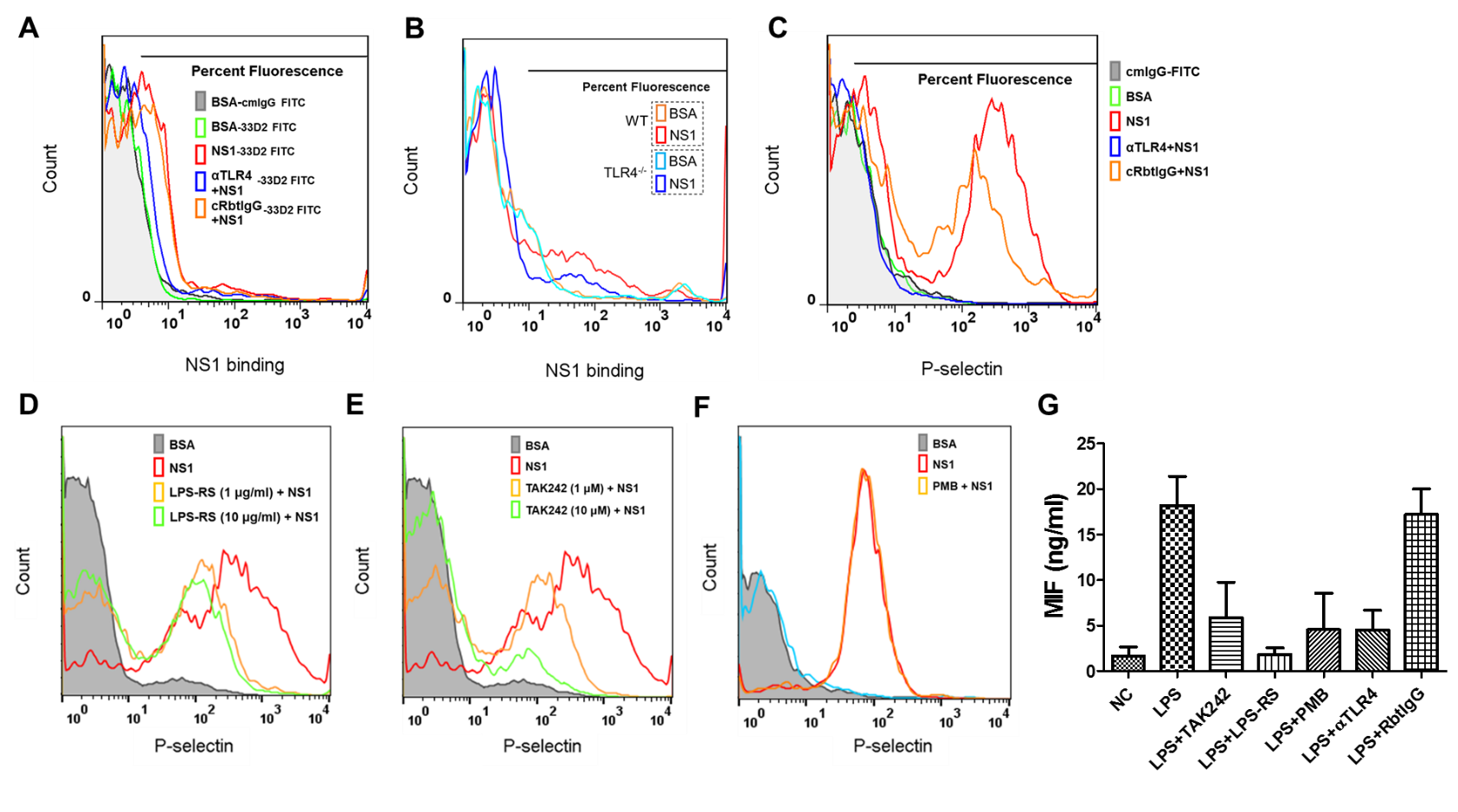
**S8 Fig. Representative plots for flow cytometry analysis of Figure 4 and the inhibitory effects of inhibitors. (A-F)** The percent fluorescence of NS1 binding and P-selectin surface expression on platelets was analyzed by FACSCalibur flow cytometry. Data analysis was performed with FlowJo software (FlowJo, LLC). (G) PMA-activated THP-1 cells were pretreated with TAK242 (10 μM), LPS-Rs (10 μg/ml), αTLR4 (5 μg/ml) or control rabbit IgG (5 μg/ml) for 30 min (or cotreated with PMB (10 μg/ml)), followed by LPS (1 μg/ml) stimulation for 24 h (n=3 per group). Cell supernatants were collected, and the concentrations of MIF in the cell supernatants were determined by human MIF ELISA kits.
