# Supplementary material for: Dengue virus nonstructural protein 1 activates platelets via Toll-like receptor 4, leading to thrombocytopenia and hemorrhage
Source: PLoS Pathog. 2019 Apr 22;15(4):e1007625. doi: 10.1371/journal.ppat.1007625 (PMC6497319; doi:10.1371/journal.ppat.1007625)
Supplement: S9 Fig — The binding of DENV NS1 to TLR4, TLR2, His-taq protein or BSA (5 μg/ml) was analyzed by ELISA, as described in the Methods. (DOCX) [file ppat.1007625.s009.docx]

 **S9 Fig. DENV NS1 could interact with both TLR4 and TLR2.** The binding of DENV NS1 to TLR4, TLR2, His-taq protein or BSA (5 μg/ml) was analyzed by ELISA, as described in the Methods.
