# Supplementary material for: Dengue virus nonstructural protein 1 activates platelets via Toll-like receptor 4, leading to thrombocytopenia and hemorrhage
Source: PLoS Pathog. 2019 Apr 22;15(4):e1007625. doi: 10.1371/journal.ppat.1007625 (PMC6497319; doi:10.1371/journal.ppat.1007625)
Supplement: S10 Fig — Human-isolated platelets were stimulated with different concentrations of LPS or DENV NS1 (10 μg/ml) for 1 h (n = 5). The percent fluorescence of P-selectin surface expression on platelets was analyzed by FACSCalibur flow cytometry, and the data analysis was performed with FlowJo software (FlowJo, LLC). *P<0.05, **P<0.01; Kruskal-Wallis ANOVA (panel B). (DOCX) [file ppat.1007625.s010.docx]

**
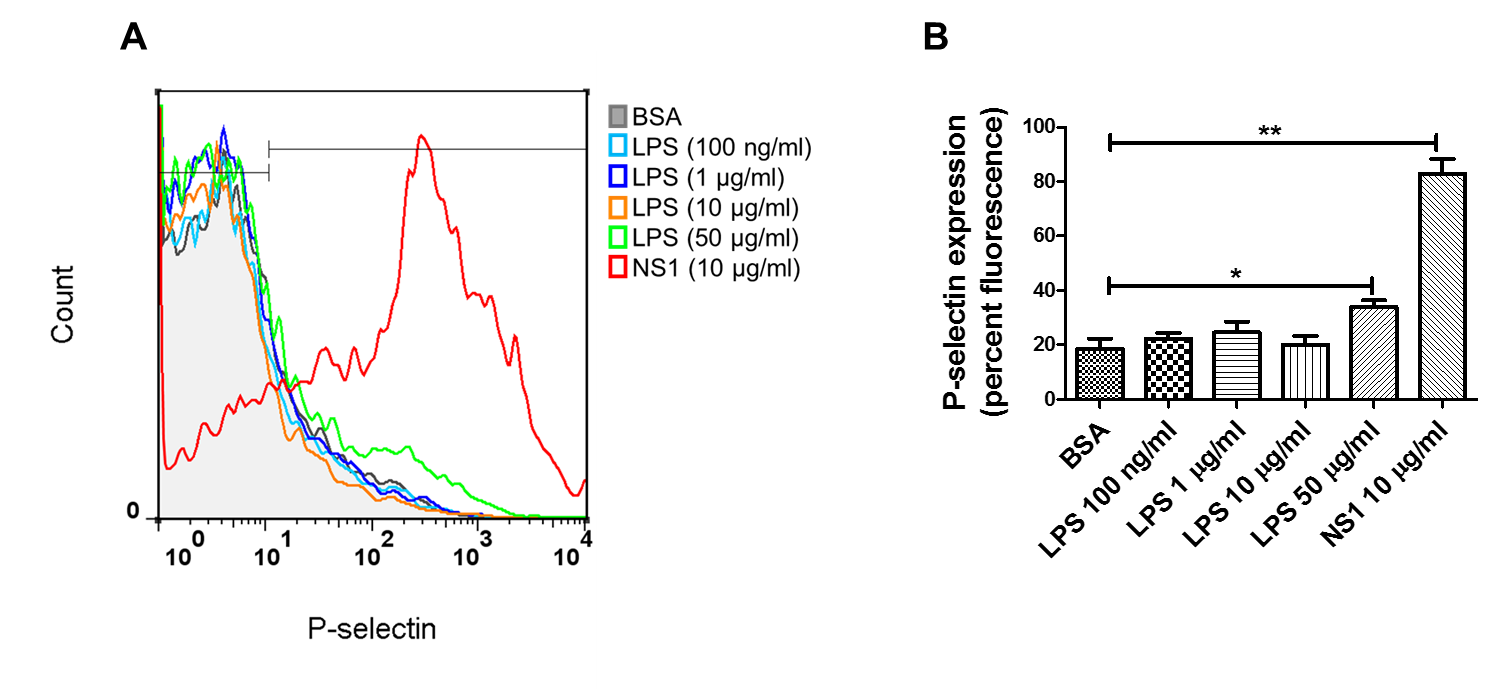
S10 Fig. LPS at a high dose could induce platelet activation.** Human-isolated platelets were stimulated with different concentrations of LPS or DENV NS1 (10 μg/ml) for 1 h (n=5). The percent fluorescence of P-selectin surface expression on platelets was analyzed by FACSCalibur flow cytometry, and the data analysis was performed with FlowJo software (FlowJo, LLC). *P<0.05, **P<0.01; Kruskal-Wallis ANOVA (panel B).
