# Supplementary material for: Dengue virus nonstructural protein 1 activates platelets via Toll-like receptor 4, leading to thrombocytopenia and hemorrhage
Source: PLoS Pathog. 2019 Apr 22;15(4):e1007625. doi: 10.1371/journal.ppat.1007625 (PMC6497319; doi:10.1371/journal.ppat.1007625)
Supplement: S11 Fig — (A)(B) Human-isolated platelets were stimulated with BSA, DENV NS1 (10 μg/ml) or thrombin (0.1 U/ml) for the indicated time, and the ADP in the supernatant was measured by ADP assay kit (ab83359, Abcam, Cambridge, UK). (C) PRP was treated with BSA or DENV NS1 (10 μg/ml) (cotreated with or without 1 μM BPTU or 1 μM Clopidogrel (both are ADP receptor inhibitors, Sigma-Aldrich) for 1 h and stimulated with ADP (2.5 μM). The light transmission of PRP was measured in a Chrono-log aggregometer. (DOCX) [file ppat.1007625.s011.docx]

**
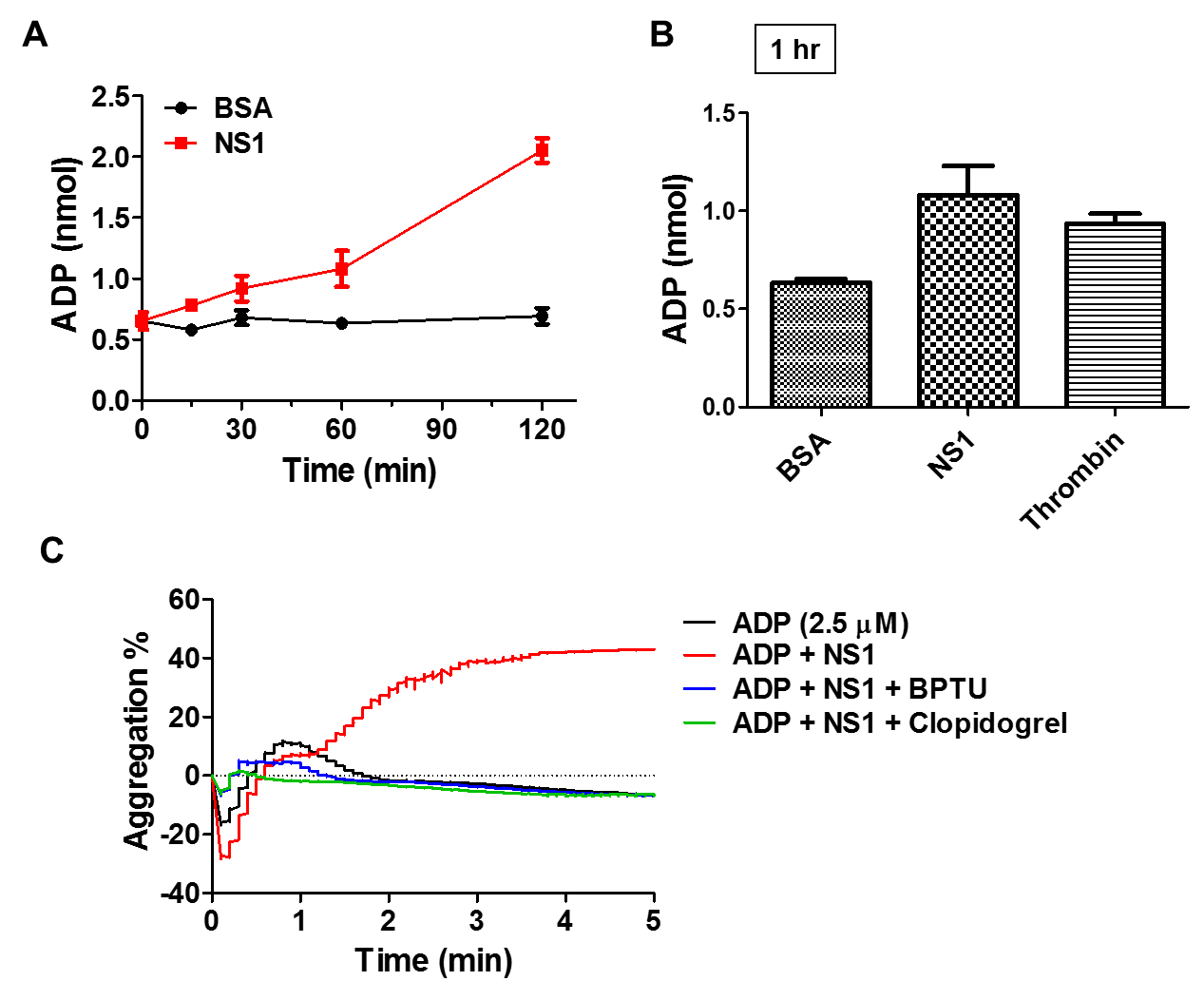
 S11 Fig. DENV NS1 induces platelets to secrete ADP. (A)(B)** Human-isolated platelets were stimulated with BSA, DENV NS1 (10 μg/ml) or thrombin (0.1 U/ml) for the indicated time, and the ADP in the supernatant was measured by ADP assay kit (ab83359, Abcam, Cambridge, UK). **(C)** PRP was treated with BSA or DENV NS1 (10 μg/ml) (cotreated with or without 1 μM BPTU or 1 μM Clopidogrel (both are ADP receptor inhibitors, Sigma-Aldrich) for 1 h and stimulated with ADP (2.5 μM). The light transmission of PRP was measured in a Chrono-log aggregometer.
