# Supplementary material for: Dengue virus nonstructural protein 1 activates platelets via Toll-like receptor 4, leading to thrombocytopenia and hemorrhage
Source: PLoS Pathog. 2019 Apr 22;15(4):e1007625. doi: 10.1371/journal.ppat.1007625 (PMC6497319; doi:10.1371/journal.ppat.1007625)
Supplement: S12 Fig — HUVEC monolayers were coincubated with washed NS1-activated platelets for the indicated time, and the relative endothelial permeability was assessed by a Transwell permeability assay, as described in the Methods. (DOCX) [file ppat.1007625.s012.docx]

 **S12 Fig. DENV NS1-activated platelets trigger endothelial hyperpermeability.** HUVEC monolayers were coincubated with washed NS1-activated platelets for the indicated time, and the relative endothelial permeability was assessed by a Transwell permeability assay, as described in the Methods.
