# Supplementary material for: Dengue virus nonstructural protein 1 activates platelets via Toll-like receptor 4, leading to thrombocytopenia and hemorrhage
Source: PLoS Pathog. 2019 Apr 22;15(4):e1007625. doi: 10.1371/journal.ppat.1007625 (PMC6497319; doi:10.1371/journal.ppat.1007625)
Supplement: S13 Fig — (A) For the phagocytosis assay, washed NS1-activated platelets (1x10^7^) were cultured with THP-1 cells for 4 h and were concentrated onto the slide using cytospin. After fixation and permeabilization, the cells were stained with CD61 (green, a marker of platelets) and CD14 (red, a marker of monocytes/macrophages) and examined by confocal microscopy. The yellow dots represent the engulfed platelets. (B)(C) THP-1 cells (5x10^5^) were incubated with washed NS1-activated platelets (1x10^7^) for the indicated time (positive control: PMA-treated THP-1 cells for 48 h). After incubation, MCP-1 mRNA expression and adherence of THP-1 cells to plates were determined. (DOCX) [file ppat.1007625.s013.docx]

**
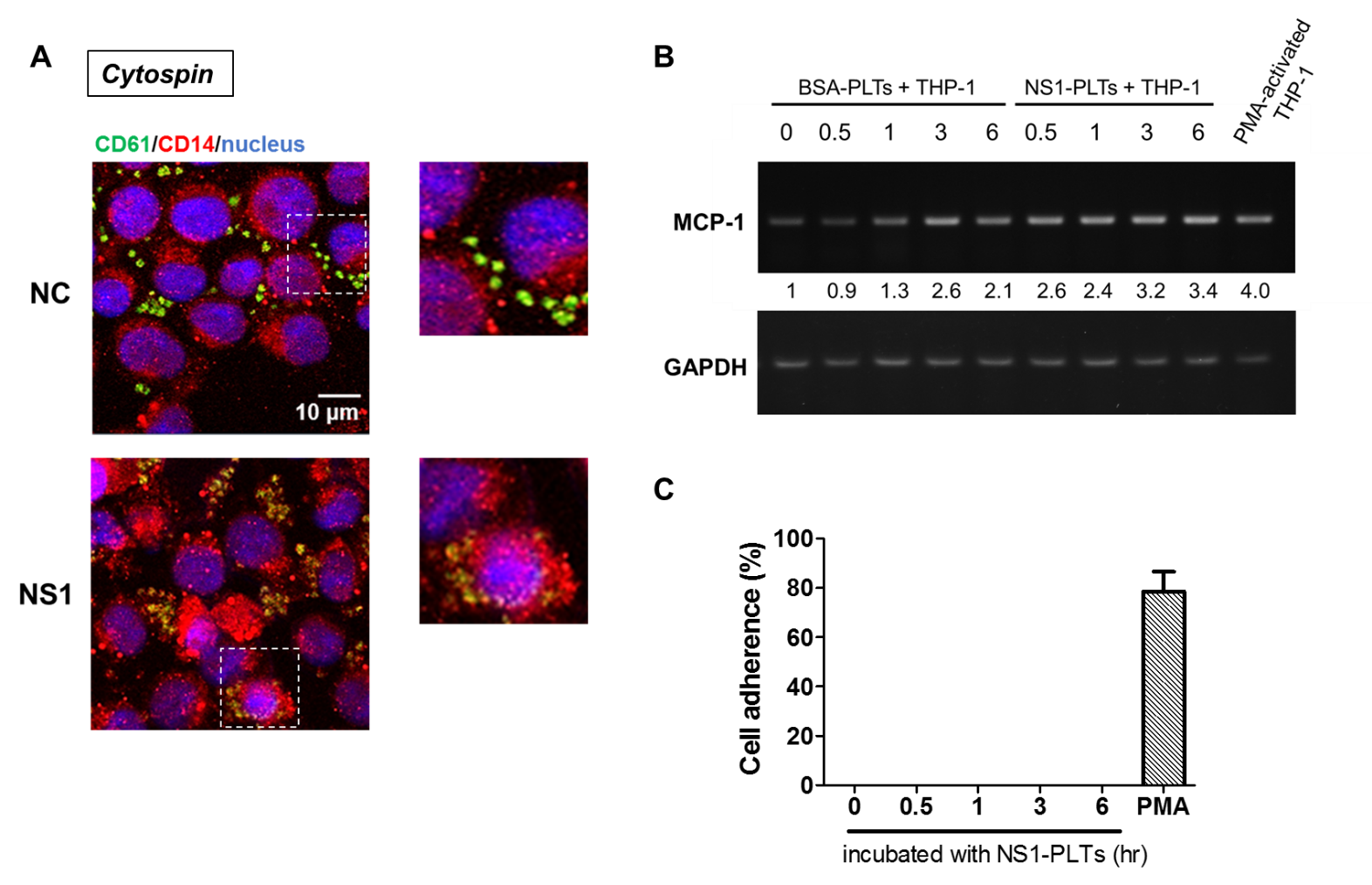
S13 Fig. THP-1 activation and phagocytosis induced by coculture with DENV NS1-activated platelets**. **(A)** For the phagocytosis assay, washed NS1-activated platelets (1x10^7^) were cultured with THP-1 cells for 4 h and were concentrated onto the slide using cytospin. After fixation and permeabilization, the cells were stained with CD61 (green, a marker of platelets) and CD14 (red, a marker of monocytes/macrophages) and examined by confocal microscopy. The yellow dots represent the engulfed platelets. **(B)(C)** THP-1 cells (5x10^5^) were incubated with washed NS1-activated platelets (1x10^7^) for the indicated time (positive control: PMA-treated THP-1 cells for 48 h). After incubation, MCP-1 mRNA expression and adherence of THP-1 cells to plates were determined.

.
