# Supplementary material for: Dengue virus nonstructural protein 1 activates platelets via Toll-like receptor 4, leading to thrombocytopenia and hemorrhage
Source: PLoS Pathog. 2019 Apr 22;15(4):e1007625. doi: 10.1371/journal.ppat.1007625 (PMC6497319; doi:10.1371/journal.ppat.1007625)
Supplement: S14 Fig — The virus titer was determined by fluorescent focus assay, and the NS1 concentration was analyzed by (A) NS1 enzyme-linked immunosorbent assay and (B) Western blotting after the experiment. (DOCX) [file ppat.1007625.s014.docx]

**
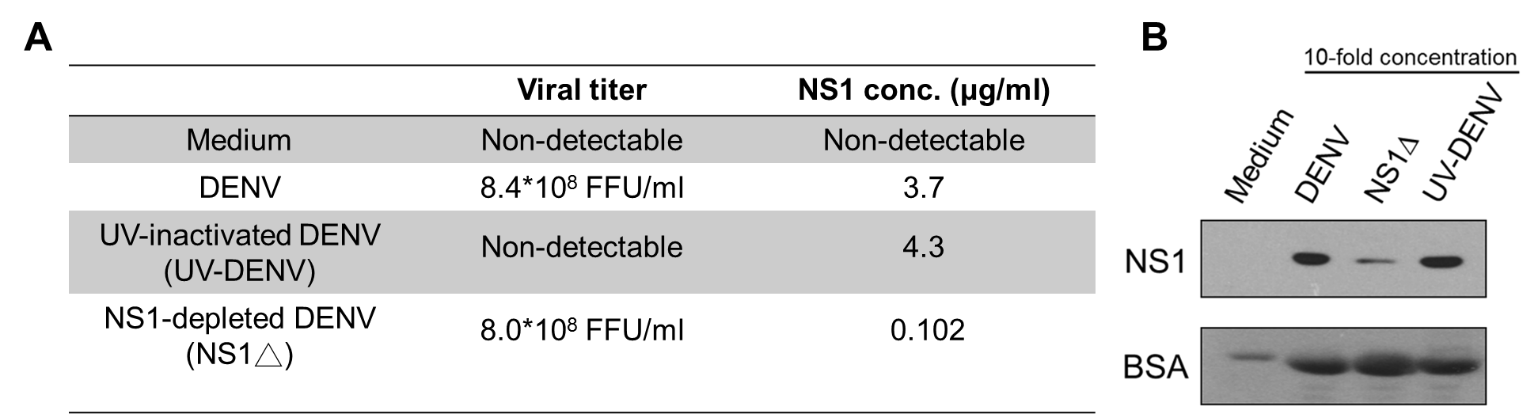
S14 Fig. Viral titer and NS1 concentrations in different conditions of DENV supernatant.** The virus titer was determined by fluorescent focus assay, and the NS1 concentration was analyzed by **(A)** NS1 enzyme-linked immunosorbent assay and **(B)** Western blotting after the experiment.

.
