# Supplementary figures and images for: Dengue virus nonstructural protein 1 activates platelets via Toll-like receptor 4, leading to thrombocytopenia and hemorrhage
Source: PLoS Pathog. 2019 Apr 22;15(4):e1007625. doi: 10.1371/journal.ppat.1007625 (PMC6497319; doi:10.1371/journal.ppat.1007625)

**
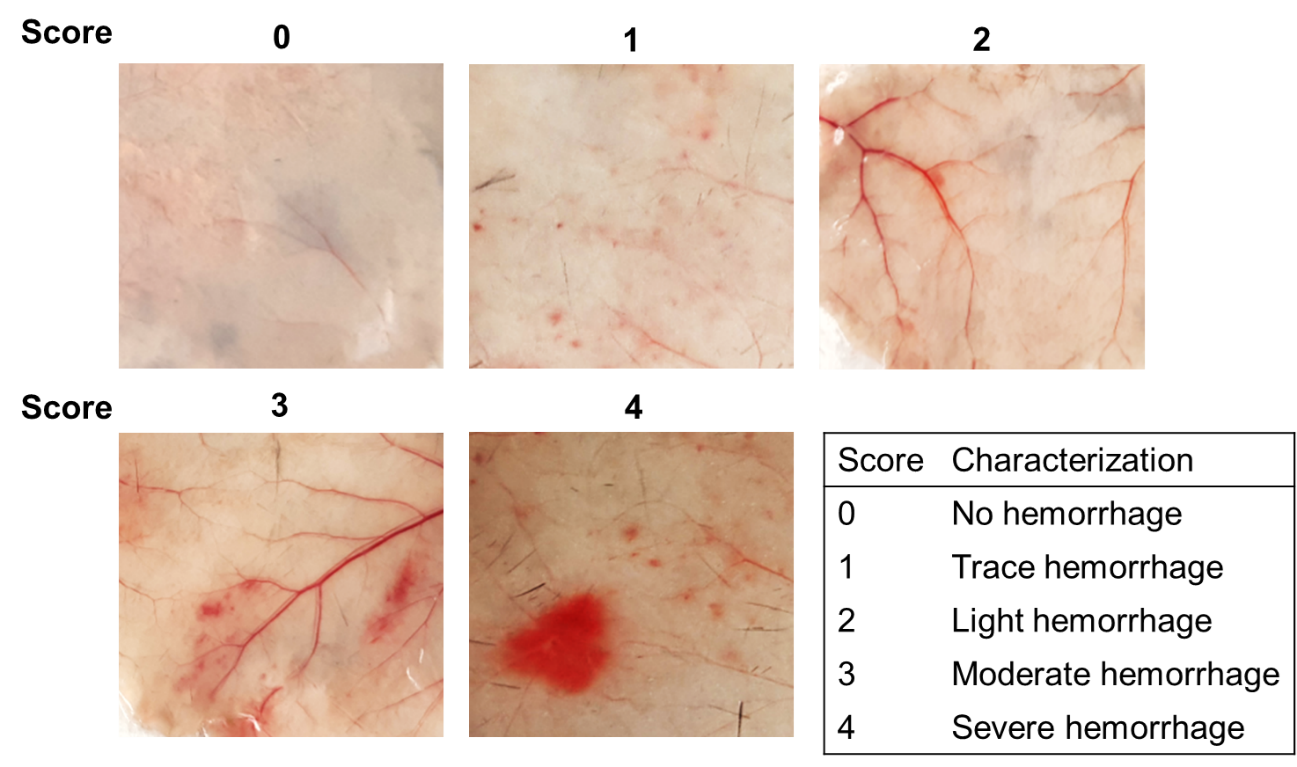
 S15 Fig. Classification of skin hemorrhage into four grades according to hemorrhage severity.**

Supplement: S15 Fig — (DOCX) [file ppat.1007625.s015.docx]
