# Supplementary material for: Dengue virus nonstructural protein 1 activates platelets via Toll-like receptor 4, leading to thrombocytopenia and hemorrhage
Source: PLoS Pathog. 2019 Apr 22;15(4):e1007625. doi: 10.1371/journal.ppat.1007625 (PMC6497319; doi:10.1371/journal.ppat.1007625)
Supplement: S16 Fig — C3H/HeN mice (n = 5) were inoculated with DENV (2x10^8^ PFU/mouse), UV-inactivated DENV or NS1-depleted DENV on the upper back. Mouse sera were collected at 3 days after inoculation, and the NS1 secretion level in mice was analyzed by NS1 quantitative ELISA. *P<0.05, **P<0.01; Kruskal-Wallis ANOVA. (DOCX) [file ppat.1007625.s016.docx]

**
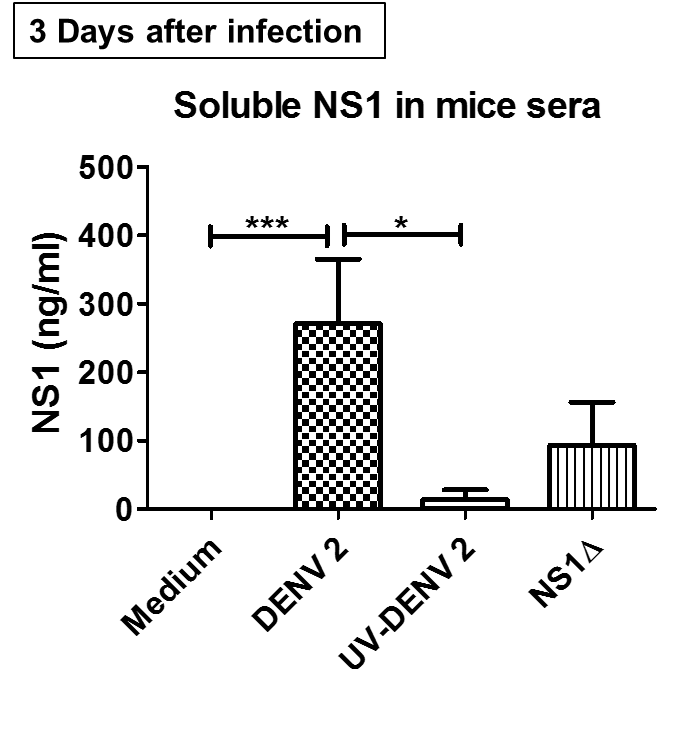
**

**S16 Fig. NS1 secretion in sera of DENV 2-infected mice.** C3H/HeN mice (n=5) were inoculated with DENV (2x10^8^ PFU/mouse), UV-inactivated DENV or NS1-depleted DENV on the upper back. Mouse sera were collected at 3 days after inoculation, and the NS1 secretion level in mice was analyzed by NS1 quantitative ELISA. *P<0.05, **P<0.01; Kruskal-Wallis ANOVA.
